# Reversible spin-optical interface in luminescent organic radicals

**DOI:** 10.1038/s41586-023-06222-1

**Published:** 2023-08-16

**Authors:** Sebastian Gorgon, Kuo Lv, Jeannine Grüne, Bluebell H. Drummond, William K. Myers, Giacomo Londi, Gaetano Ricci, Danillo Valverde, Claire Tonnelé, Petri Murto, Alexander S. Romanov, David Casanova, Vladimir Dyakonov, Andreas Sperlich, David Beljonne, Yoann Olivier, Feng Li, Richard H. Friend, Emrys W. Evans

**Affiliations:** 1 Cavendish Laboratory, University of Cambridge, Cambridge, UK; 2 Centre for Advanced Electron Spin Resonance, Department of Chemistry, University of Oxford, Inorganic Chemistry Laboratory, Oxford, UK; 3 State Key Laboratory of Supramolecular Structure and Materials, College of Chemistry, Jilin University, Changchun, P. R. China; 4 Experimental Physics VI, Faculty of Physics and Astronomy, University of Würzburg, Würzburg, Germany; 5 Laboratory for Computational Modelling of Functional Materials, Namur Institute of Structured Matter, University of Namur, Namur, Belgium; 6 Donostia International Physics Centre, Donostia, Spain; 7 Yusuf Hamied Department of Chemistry, University of Cambridge, Cambridge, UK; 8 Department of Chemistry, University of Manchester, Manchester, UK; 9 Laboratory for Chemistry of Novel Materials, University of Mons, Mons, Belgium; 10 Department of Chemistry, Swansea University, Swansea, UK; 11 Present Address: Cavendish Laboratory, University of Cambridge, Cambridge, UK

**Keywords:** Chemical physics, Excited states, Molecular electronics, Quantum physics

## Abstract

Molecules present a versatile platform for quantum information science^1,2^ and are candidates for sensing and computation applications^3,4^. Robust spin-optical interfaces are key to harnessing the quantum resources of materials^5^. To date, carbon-based candidates have been non-luminescent^6,7^, which prevents optical readout via emission. Here we report organic molecules showing both efficient luminescence and near-unity generation yield of excited states with spin multiplicity *S* > 1. This was achieved by designing an energy resonance between emissive doublet and triplet levels, here on covalently coupled tris(2,4,6-trichlorophenyl) methyl-carbazole radicals and anthracene. We observed that the doublet photoexcitation delocalized onto the linked acene within a few picoseconds and subsequently evolved to a pure high-spin state (quartet for monoradical, quintet for biradical) of mixed radical–triplet character near 1.8 eV. These high-spin states are coherently addressable with microwaves even at 295 K, with optical readout enabled by reverse intersystem crossing to emissive states. Furthermore, for the biradical, on return to the ground state the previously uncorrelated radical spins either side of the anthracene shows strong spin correlation. Our approach simultaneously supports a high efficiency of initialization, spin manipulations and light-based readout at room temperature. The integration of luminescence and high-spin states creates an organic materials platform for emerging quantum technologies.

## Abstract (web summary)

We report organic molecules showing both efficient luminescence and near-unity generation yield of excited states with high spin multiplicity, simultaneously supporting a high efficiency of initialization, spin manipulations and light-based readout at room temperature.

## Main

Considerable progress has been made towards designing molecular systems fulfilling the DiVincenzo criteria for practical qubits^8^. Optical addressability has been demonstrated in organometallic complexes with triplet ground states at liquid helium temperatures^9^. Related complexes show impressive spin coherence times, reaching the microsecond range at room temperature^10^. Structures without metal atoms can be more immune to decoherence^11^, and such fully organic molecules have been used in several demonstrations of quantum effects^12–14^.

Radical organic molecules contain unpaired electrons that can be stabilized by chemical design. Advances have been made using non-luminescent radicals that are covalently attached to chromophores, and such structures can support excited states with high spin multiplicity^6,7^. The presence of a radical can enhance the rate of intersystem crossing (ISC), leading to accumulation of chromophore triplet states^15^. If exchange between the triplet (*S* = 1) and radical (*S* = 1/2) spin is larger than all other magnetic interactions, a distinct quartet (‘trip-quartet’, *S* = 3/2) and doublet (‘trip-doublet’, *S* = 1/2) state pair forms^16^. If a second radical is additionally coupled, a quintet (‘trip-quintet’, *S* = 2) state can be achieved^17^. High-spin states allow the building of dense architectures with multiple qubits hosted within a single manifold of spin sublevels^18^. Such multilevel qubits, termed qudits, offer scaleability advantages in quantum computation^19^. The qudit behaviour of quartet states was recently demonstrated in PDI-TEMPO at 80 K (ref. ^20^). However, current high-spin structures have large (about 1 eV) energy gaps between the photogenerated chromophore singlet state and the triplet manifold^6,17,21^. Critically, this prevents reverse ISC (RISC) to a luminescent state. Thus all organic high-spin systems with *S* > 1 to date are non-emissive, which makes optical readout impossible.

Whereas most stable radicals are non-emissive, there is now a class of luminescent radicals that offer fully spin-allowed emission within the doublet manifold^22^. The set of available molecular structures and their optical wavelength range is expanding^23,24^. Record efficiencies for deep-red and infrared light-emitting diodes were recently reached in tris(2,4,6-trichlorophenyl) methyl (TTM) radicals linked to carbazole electron donors^25^.

By utilization of a doublet (D_1_) level with substantial oscillator strength for absorption and emission, we can avoid excitation via the singlet state in radical-chromophore structures. In our designs we bring the triplet (T_1_) level into energy resonance with a D_1_ level on a luminescent radical. Eliminating the energy gap between photogenerated and high-spin states allows interconversion in either direction. This makes it possible to initialize and optically read out high-spin states with *S* > 1 in organic molecules.

## Optical properties

**Fig. 1:**
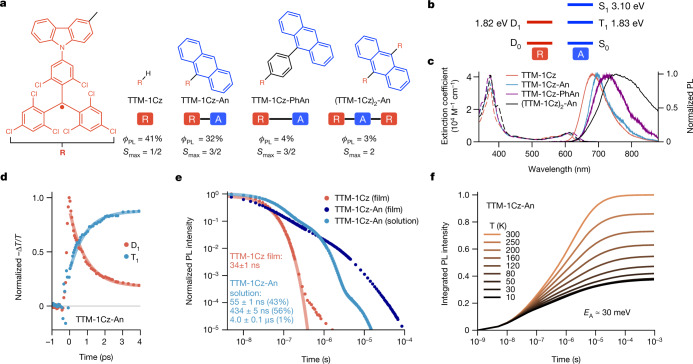
Luminescent radical-acene molecular system. **a**, Molecules featured in this study, their dilute toluene solution PLQE (*ϕ*_PL_) and the highest spin quantum number of their excited state (*S*_max_). **b**, Energy levels for TTM-1Cz and anthracene, extracted from emission data in separated molecules, showing the D_1_–T_1_ energy alignment. **c**, Steady-state absorption (dashed lines) and photoluminescence (solid lines) spectra obtained following 532 nm excitation in 200 μM toluene solution at room temperature. Anthracene-linked compounds show a small red-shift relative to TTM-1Cz. **d**, Ultrafast transient absorption kinetics of dilute toluene TTM-1Cz-An solution following a 600 nm pulse at 295 K extracted from photoinduced absorption features. Solid lines show 0.92 ps lifetime fits. **e**, Emission kinetics of 5% in PMMA films and solutions of TTM-1Cz and TTM-1Cz-An at 295 K following 532 nm excitation. **f**, Temperature dependence of integrated emission intensity of 5% TTM-1Cz-An in PMMA films following 520 nm excitation, showing temperature activation of radical emission. *E*_A_, activation energy.

We used TTM-1Cz as a luminescent radical unit ‘R’ because it shows 41% photoluminescence quantum efficiency (PLQE) for red emission in dilute toluene solution (Fig. 1a)^26^. Its small size allows proximity between the radical and chromophore. To achieve doublet–triplet energy resonance involving the emissive excited state with E(D_1_) = 1.82 eV, we selected anthracene with E(T_1_) = 1.83 eV (Fig. 1b) linked at its 9-position to the para-position of the TTM-1Cz carbazole, thus preserving through-bond conjugation (Supplementary Information Section 1). We prepared two ‘R-A’ monoradical structures (TTM-1Cz-An, and TTM-1Cz-PhAn with a bridging phenyl ring) and an ‘R-A-R’ biradical (TTM-1Cz)_2_-An. At room temperature their absorption spectra were only weakly modified and their photoluminescence (PL) showed a red-shift relative to TTM-1Cz (Fig. 1c). The PLQE in toluene solution was 32% for TTM-1Cz-An, 4% for TTM-1Cz-PhAn and 3% for (TTM-1Cz)_2_-An.

**Extended Data Fig. 1:**
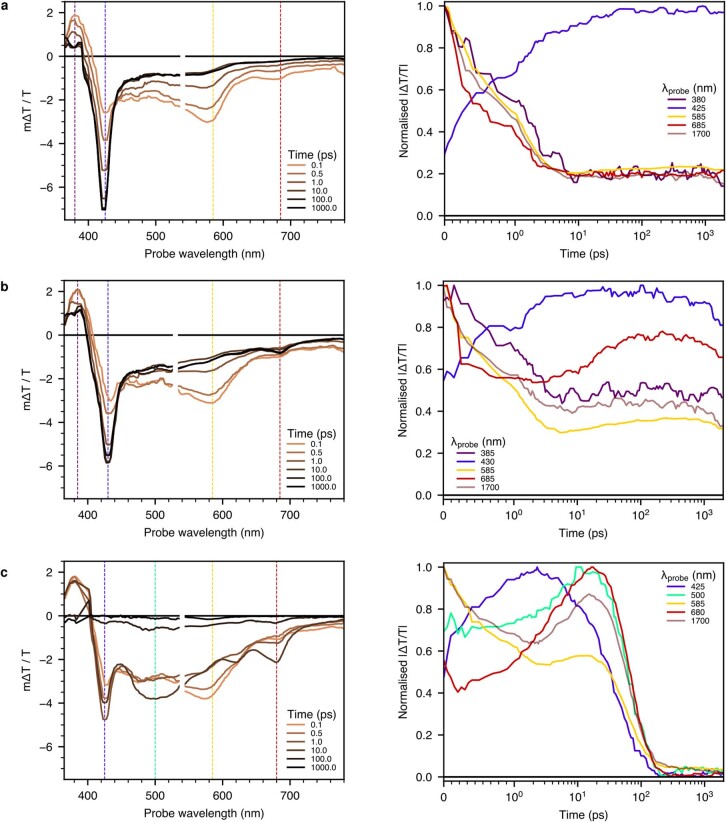
TTM-1Cz-An transient optical absorption. Spectral (left) and kinetic (right) TA slices of 200 μM solutions in **a**, cyclohexane, **b**, toluene, and **c**, 2-methyltetrohydrofuran (MeTHF). In kinetics, time axis scale is linear until 1 ps, and logarithmic thereafter; and absolute values of the TA signal normalised to their peak are shown. Excitation at 600 nm with fluences of 40 μJ cm^−2^, matched between 360–580 and 530–780 nm probe region experiments for each plot. Solvatochromism observed after ca. 5 ps, i.e. following rapid energy transfer. CT signature at 685 nm absent in cyclohexane (least polar). In MeTHF (most polar) non-radiative decay occurs after the emergence of the CT band.

We performed transient optical absorption spectroscopy to establish the mechanism of interaction between radical and linked anthracene. We selectively excited the radical using a 600 nm pulse, well below the anthracene singlet absorption onset. In dilute solutions of R-A and R-A-R we observed rapid decay of D_1_ photoinduced absorption (PIA) and a matching rise of T_1_ PIA, indicating transfer of excitation from TTM-1Cz to the anthracene with local T_1_ character (Extended Data Fig. 1). This occurred with an ultrafast lifetime of 0.9 ± 0.2 ps in TTM-1Cz-An (Fig. 1d), 5.6 ± 0.6 ps in TTM-1Cz-PhAn and 0.7 ± 0.2 ps in (TTM-1Cz)_2_-An (Supplementary Fig. 11).

We focused on TTM-1Cz-An dynamics because it is the most emissive material. The prompt transient optical absorption spectra show that partial local triplet character was already present within our 150 fs time resolution. When solvent polarity was increased from cyclohexane to toluene there were no changes in rapid energy transfer dynamics and we extracted a 93% yield of T_1_ generation (Supplementary Fig. 12). Subsequently a new PIA near 685 nm appeared at around 30 ps, which we assigned to an intramolecular charge-transfer (CT) state because its spectral position matched the anthracene radical cation^27^. The CT state population peaked at around 100 ps in toluene solution. In the more polar 2-methyltetrahydrofuran we observed complete non-radiative decay within 1 ns, likely via a low-lying CT state.

**Extended Data Fig. 2:**
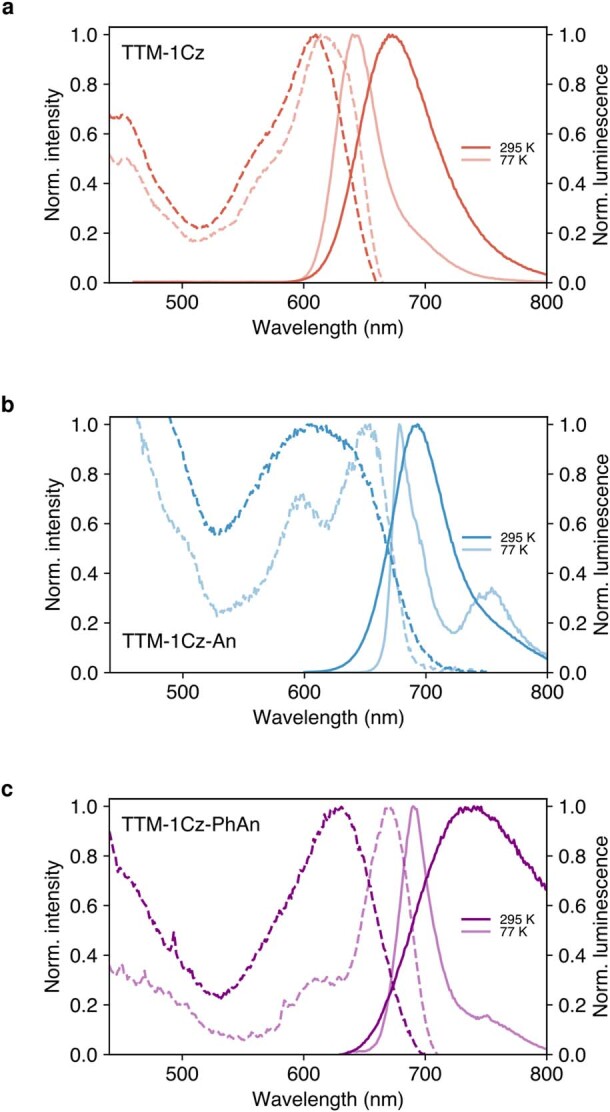
Steady state photophysics. Excitation and emission scans at 295 and 77 K in 200 μM toluene solutions of **a**, TTM-1Cz, **b**, TTM-1Cz-An, and **c**, TTM-1Cz-PhAn. Intensity monitored at luminescence maxima during excitation scans. Vibronic progression reminiscent of pure anthracene is seen in TTM-1Cz-An and TTM-1Cz-PhAn at low temperatures.

We performed time-resolved emission spectroscopy to understand how D_1_ emission is preserved despite rapid energy transfer dynamics. TTM-1Cz-An emission showed triexponential kinetics in toluene solution at room temperature (Fig. 1e), the emission line shape remaining unchanged throughout the decay (Supplementary Fig. 13). The fastest emission component (55 ± 1 ns) was twofold slower compared with the mono-exponential lifetime of TTM-1Cz in the same solvent (27 ± 1 ns). Most TTM-1Cz-An emission was delayed even further and occurred with a lifetime of 434 ± 5 ns. Because the D_1_ character was lost with near-unity yield well before the TTM-1Cz emission lifetime, all radical emission in TTM-1Cz-An was preceded by a temperature-activated process. The TTM-1Cz-An emission lineshape became more structured as temperature was lowered, resembling anthracene phosphorescence at 10 K (Supplementary Fig. 14). A vibronic progression was present in both excitation and emission scans at 77 K, similar to that of anthracene triplets (Extended Data Fig. 2)^28^. These features could signify a partial T_1_-like character of the photogenerated state.

**Extended Data Fig. 3:**
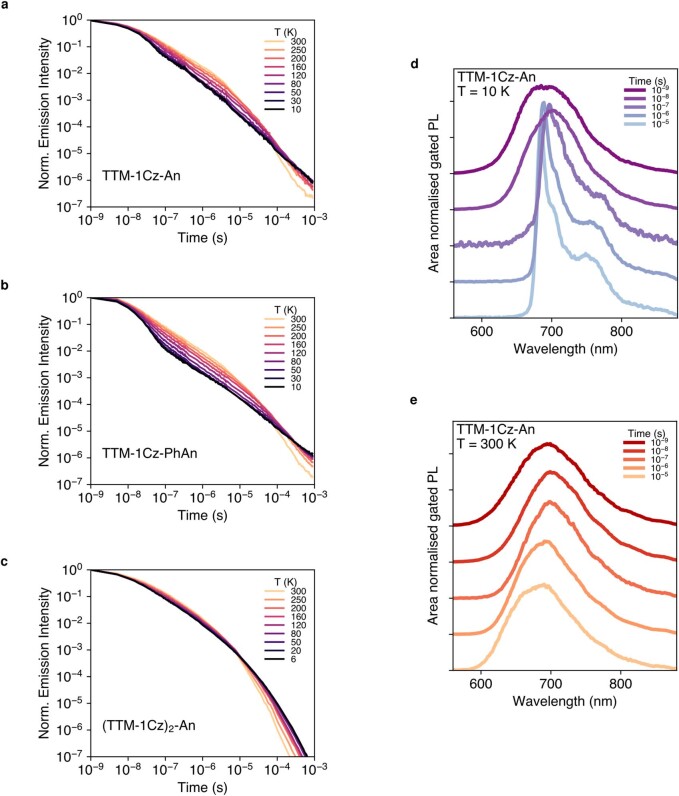
Temperature dependent transient photoluminescence. Normalised emission kinetics in 5% PMMA films of **a**, TTM-1Cz-An,**b**, TTM-1Cz-PhAn, and **c**, (TTM-1Cz)_2_-An. All materials show temperature-activated delayed emission. **d**, Time gated emission spectra in 5% TTM-1Cz-An in PMMA films at 10 K, and **e**, 300 K. All measurements performed in vacuum after excitation at 520 nm with fluence of 26 μJ cm^−2^ (for R-A) or 530 nm with fluence of 8.7 μJ cm^−2^ (for R-A-R).

We performed temperature-dependent, time-resolved emission spectroscopy in dilute poly(methyl methacrylate) (PMMA) films (Extended Data Fig. 3). We found that total emission intensity strongly increased with temperature (Fig. 1f), in contrast to the temperature-independent intensity and rate of D_1_ emission in TTM-1Cz (Supplementary Fig. 15). Using an Arrhenius-type model we estimated the activation energy for emission in TTM-1Cz-An at approximately 30 meV. All R-A and R-A-R showed temperature-activated emission and structured phosphorescence at low temperatures (Supplementary Fig. 16). The activation energy for delayed emission in the (TTM-1Cz)_2_-An biradical was approximately 15 meV.

## Spin properties

**Fig. 2:**
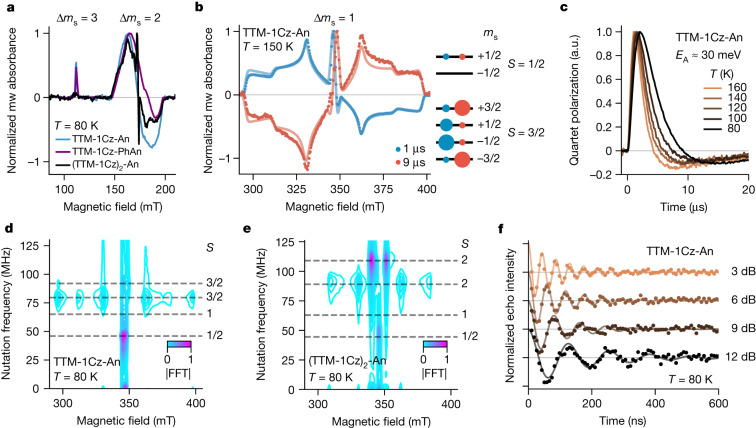
ESR on high-spin states. **a**, Transient half-field continuous-wave X-band (9.7 GHz) ESR spectra at 80 K following 600 nm excitation, showing Δ*m*_s_ = 3 and Δ*m*_s_ = 2 transitions in frozen toluene solution of R-A and a patterned Δ*m*_s_ = 2 in R-A-R. **b**, Transient full-field continuous-wave X-band ESR Δ*m*_s_ = 1 spectra \ (points) and simulations (lines) of TTM-1Cz-An frozen toluene solution at 150 K following 600 nm excitation, and schematic population patterns extracted from the model. Circle size is proportional to the sublevel population at early (blue) and late (red) times. **c**, Temperature dependence of TTM-1Cz-An quartet (331.5 mT) polarization inversion. **d**, Transient nutation at 80 K of dilute frozen toluene solution of TTM-1Cz-An following 600 nm excitation, indicating quartet (*S* = 3/2) state formation. FFT, fast Fourier transform. **e**, Transient nutation at 80 K of dilute frozen toluene solution of (TTM-1Cz)_2_-An following 600 nm excitation, showing quintet (*S* = 2) multiplicity of the polarized signal. **f**, Rabi oscillations on the TTM-1Cz-An quartet (331.5 mT) at 80 K as a function of microwave power. a.u., arbitrary units.

**Extended Data Fig. 4:**
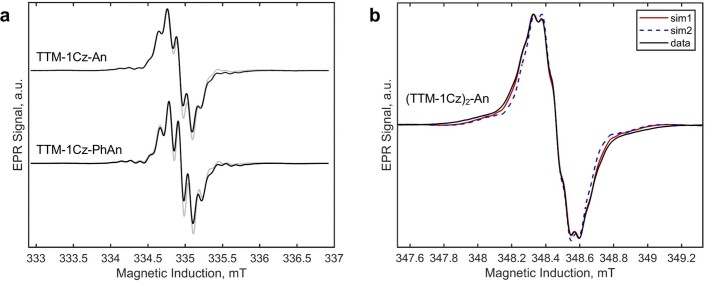
Ground state ESR. **a**, Room temperature X-band cw-ESR of 200 µM toluene solutions of TTM-1Cz-An and TTM-1Cz-PhAn in the dark confirming their spin doublet ground states. The black traces are data, and the grey lines are simulations. The respective acquisition parameter values were microwave frequencies 9.3923 & 9.3927 GHz, field modulations of 5 & 20 μT, microwave powers of 200 & 100 nW, both being non-saturating. Simulation employed experimental parameter values with S = 1/2, g = 2.00355 (±0.00005), six *A*_iso_(^1^H) = 0.121 mT, one *A*_iso_(C_nat. abund._) = 2.7 mT, and six *A*_iso_(C_nat. abund._) = 0.55 mT. **b**, cw-ESR at X-band of 200 µM toluene solution of (TTM-1Cz)_2_-An at 295 K, shown in the black trace with two simulations. The acquisition microwave frequency was 9.7715 GHz with power of 2 µW, the field modulation amplitude was 2 µT. As *J* > *A*, the hyperfine values are divided by a factor of two compared to TTM-1Cz-An. The best fit is provided by increasing the number of contributing protons with *A*(^1^H) = 0.06 mT from twice the TTM-1Cz-An simulation in panel a (12 protons, sim2) to 14 protons (sim1). The conformational degrees of freedom in solution provide a range of exchange values, leading to an average in excess of the apparent hyperfine values.

**Extended Data Fig. 5:**
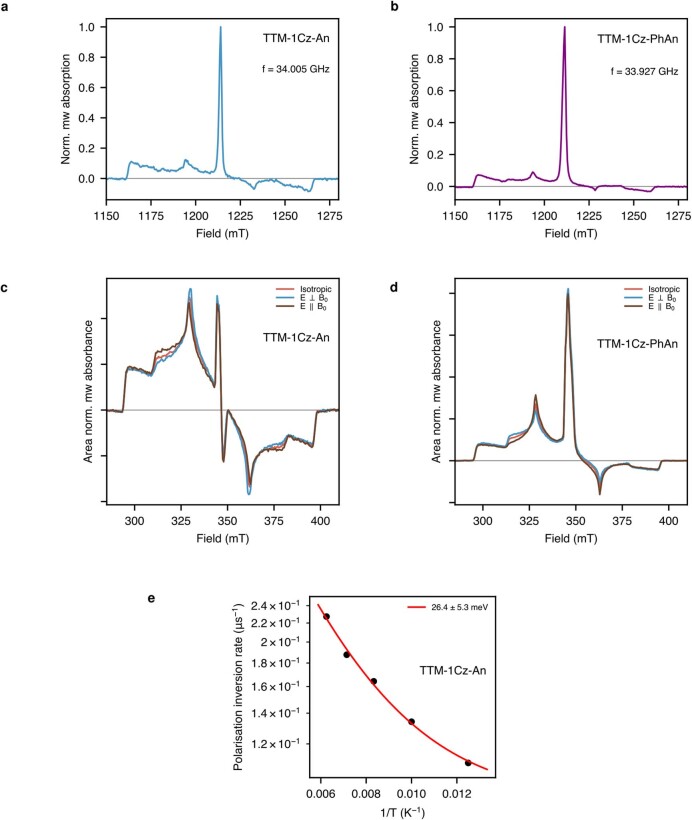
R-A trESR. **a**, Q-band FF trESR on frozen 200 μM toluene solutions, 1–4 μs after excitation with 0.4 mJ 600 nm pulses. The lineshape is analogous to the X-band FF spectra, with no level crossings detected at fields up to 1.3 T. **b**, Magnetophotoselection in X-band FF trESR. Dilute toluene solutions at 80 K following 0.7 mJ 600 nm excitation at 40 dB microwave attenuation. Signals normalized by total area. No selection observed for z transitions, and opposite dependence for TTM-1Cz-An and TTM-1Cz-PhAn seen for x/y transitions. **c**, Arrhenius plot of quartet polarisation inversion dynamics in TTM-1Cz-An.

Our optical experiments show rapid generation of a local triplet character excited state followed by temperature-activated emission with D_1_ character. We used electron spin resonance (ESR) to probe the spin properties of the states involved in this mechanism. Continuous-wave ESR in the dark showed a narrow signal centred at *g* = 2.0036 for all four molecules studied (Extended Data Fig. 4), characteristic of a TTM D_0_ transition. We performed transient continuous-wave ESR (trESR) to track the excited-state sublevel dynamics following selective D_1_ excitation with a pulsed laser^29^. At X-band in the half-field region, R-A showed signals centred at *g* = 4.04 and *g* = 6.20 (Fig. 2a). These are first-order forbidden transitions with changes in projection spin quantum numbers of Δ*m*_s_ = 2 and Δ*m*_s_ = 3, respectively, in which the latter gives clear evidence of probing a quartet state (Supplementary Fig. 17). In the full-field region, dilute frozen toluene solutions of R-A showed a broad Δ*m*_s_ = 1 signal with a width of around 103 mT, superimposed with a narrow signal at *g* = 2.00 (Fig. 2b). Signal width was reduced by a factor of 2/3 compared with that expected from anthracene triplets (roughly 152 mT)^30^. This suggests that a quartet state was formed due to strong exchange between the anthracene triplet-like wavefunction and ground-state radical spin. Absence of level crossings in trESR at Q-band indicates that the R-A quartet–doublet energy gap was at least 0.8 meV (Extended Data Fig. 5). The polarization pattern inverted at later times, with inversion occurring faster at increasing temperature (Fig. 2c).

Using a transient nutation pulse sequence we can directly confirm the spin multiplicity of the sublevels involved in ESR transitions^31^. In the dark, only the doublet nutation frequency (*ω*_0_) is detected for both R-A. Following 600 nm light excitation we found no contribution from triplet transitions (*ω* = √2*ω*_0_) across the entire spectra of R-A. The broad feature is due to ‘outer’ quartet transitions (*ω* = √3*ω*_0_), and the narrow central features are due to both ‘inner’ quartet (*ω* = 2*ω*_0_) and doublet (*ω* = *ω*_0_) transitions (Fig. 2d). This confirms that the strong exchange regime exists throughout the entire molecular ensemble, regardless of conformational effects.

The presence of strong exchange in R-A indicates that the anthracene–carbazole linkage supports significant wavefunction delocalization. In the symmetrically substituted (TTM-1Cz)_2_-An biradical, the full-field trESR spectrum was further narrowed (Supplementary Fig. 18). Its width of about 77 mT is consistent with the formation of a quintet state in the strong exchange regime. The polarization pattern shows an ISC population mechanism, as in R-A quartets. Transient nutation confirmed that the broad spectrum of (TTM-1Cz)_2_-An was due to quintet transitions (*ω* = √6*ω*_0_ or *ω* = 2*ω*_0_), and the narrow central feature was exclusively due to a doublet transition (Fig. 2e). We did not detect any quartet or uncoupled triplet features, which shows that triplet character excitation on the anthracene in R-A-R was strongly coupled to both radical electrons within an overall four-spin state.

Turning to the most luminescent TTM-1Cz-An, we have explored Rabi oscillation experiments to quantify its potential as a molecular qudit coupled to emission^32^. We placed the quartet in a coherent superposition, which was then probed with a Hahn echo sequence (Fig. 2f). This allowed us to find the quantum fidelity ${\Omega }_{{\rm{M}}}=2{T}_{{\rm{m}}}{\omega }_{{\rm{R}}}$, where *T*_m_ is spin coherence time and *ω*_R_ is Rabi frequency. At 80 K, *T*_m_ is 1.5 ± 0.1 μs and Ω_M_ values of up to around 70 were found, comparable to quartet states in non-luminescent molecules^20^. The Ω_M_ values scale linearly with microwave (mw) power, showing that this quartet state can be placed in an arbitrary superposition.

We modelled R-A trESR spectra to track sublevel population dynamics (Extended Data Table 1). The prompt signal showed a majority population of the quartet *m*_s_ = ±1/2 sublevels at all temperatures (Fig. 2b). By fitting quartet polarization inversion times to an Arrhenius-type model we found an activation energy of 26 ± 5 meV for TTM-1Cz-An (Extended Data Fig. 5). This is in excellent agreement with the activation energy for emission found by optical spectroscopy, and confirms that D_1_ emission is preceded by RISC from the quartet state. The *m*_s_ = ±3/2 sublevels formed the majority of the quartet population following inversion, consistent with a preferential depopulation of *m*_s_ = ±1/2 sublevels during RISC.

## Luminescent R-A mechanism

**Fig. 3:**
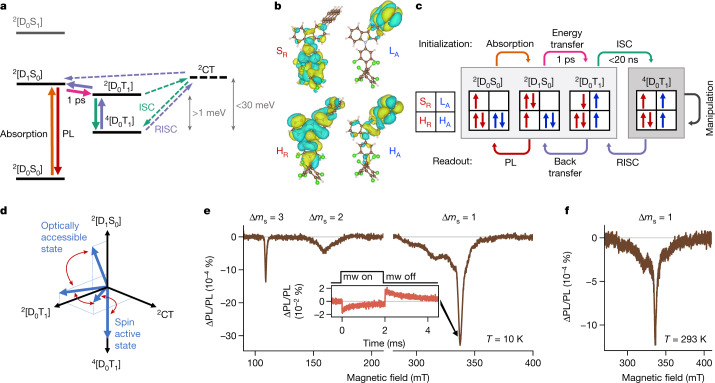
Luminescent R-A mechanism and optical readout. **a**, R-A energy level diagram showing rapid quartet state generation following light absorption by the radical. At room temperature, activation to the ^2^[D_1_S_0_] state is efficient and only radical PL is seen. **b**, Molecular orbitals of TTM-1Cz-An obtained with MCSCF calculations showing highest occupied molecular orbital (HOMO), lowest unoccupied molecular orbital (LUMO) and singly occupied molecular orbital (SOMO) from radical and acene components (H_R_, radical HOMO; S_R_, radical SOMO; H_A_, acene HOMO; L_A_, acene LUMO). **c**, Summary scheme of dominant orbital contributions during quartet formation and radical emission. **d**, Vectorial depiction of the evolution of state mixing during reversible quartet formation. Character of the photoexcited doublet state evolves from majority ^2^[D_1_S_0_] to majority ^2^[D_0_T_1_] at ultrafast timescales. If energetically accessible, the ^2^CT state may be additionally involved in doublet state mixing and thus assist the ISC toward a pure quartet state. **e**, ODMR spectra (9.4 GHz) of dilute frozen toluene solution of TTM-1Cz-PhAn under 532 nm excitation at 10 K, showing participation of the quartet state in the full-field and half-field region. Inset, trODMR of TTM-1Cz-PhAn at 10 K at 337.5 mT showing PL reduction in resonance. **f**, ODMR spectra of dilute PMMA films of TTM-1Cz-PhAn under 532 nm excitation at 293 K.

Our ESR results show that a complete description of the electronic states in R-A and R-A-R requires knowledge of their total spin multiplicity, as well as of the nature of the contributing anthracene-like and TTM-1Cz-like states (Fig. 3a). Multiconfigurational self-consistent field (MCSCF) calculations for TTM-1Cz-An (Supplementary Information Section 3) show the photogenerated state as a transition between the H_R_ and S_R_ molecular orbitals. This state is similar to the D_1_ state in TTM-1Cz but, additionally, contains some wavefunction density on the linked anthracene in the H_R_ molecular orbital (Fig. 3b). At ground-state equilibrium geometry, this state contains approximately 5% contribution on the anthracene (Supplementary Fig. 26) and lies 13 meV above states with local triplet character (Supplementary Fig. 21 and Supplementary Table 2). The calculations also show the presence of an intramolecular charge-transfer state (^2^CT) from the anthracene to the TTM-1Cz moiety that spans a broad energy range, from roughly 60 meV above the photogenerated state at the equilibrium geometry to about 20 meV below that state, for an orthogonal arrangement of the anthracene unit (Supplementary Table 5). We have developed a kinetic model for energy transfer that includes a ^2^CT intermediate (Supplementary Table 15). We computed energy transfer times spanning a 0.1–10 ps range when close to the ground-state equilibrium conformation. The calculated quartet state spin Hamiltonian parameters agree well with those extracted from modelling the ESR data (Supplementary Fig. 24 and Supplementary Table 9).

In the pure diabatic eigenstate description of R-A, absorption of light with energies below singlet anthracene band gap occurs from ^2^[D_0_S_0_] to ^2^[D_1_S_0_] ‘doub-doublet’ (Fig. 3c). This photoexcited state is energetically close to ^2^[D_0_T_1_], and rapid localization of the wavefunction onto the coupled anthracene occurs within a few picoseconds. Such high rates are possible because total spin multiplicity is conserved during ^2^[D_1_S_0_] to ^2^[D_0_T_1_] energy transfer. Subsequently the ^2^[D_0_T_1_] exciton undergoes ISC to the ^4^[D_0_T_1_] quartet state. Because the spatial wavefunctions of the ^2^[D_0_T_1_] and ^4^[D_0_T_1_] levels are nearly identical, the direct spin–orbit coupling matrix element is small (Supplementary Table 6)^33^. Direct ISC is thus unlikely to generate the high quartet yields on sub-20-ns timescales as implied by the observed emission dynamics. We consider that the ^2^CT state is close in energy to ^2,4^[D_0_T_1_] excitonic states. This is supported by solvatochromism observed in transient optical absorption and MCSCF calculations. Accessibility of a CT state might assist quartet state formation via spin–orbit coupling, which is larger for states of different character (Supplementary Table 8). The ^2^CT state can form from ^2^[D_0_T_1_] following spin-conserving electron transfer from L_A_ onto S_R_. If a spin-flip occurs during back-transfer, the ^4^[D_0_T_1_] state forms. This forward mechanism is near barrierless and supports our observations of a high yield of quartet states at temperatures between 20 and 300 K. We can estimate quartet state yield using luminescence dynamics and PLQE, together with the T_1_ yield extracted from transient optical absorption. The yield of ^4^[D_0_T_1_] states in toluene solution of TTM-1Cz-An at room temperature is approximately 73%.

The process preceding emission is temperature activated. Due to low energetic barriers present in our molecular design, only around 22% of total emission yield is lost in TTM-1Cz-An compared with TTM-1Cz at room temperature. Reforming a spin doublet state from the quartet is the rate-limiting step due to the need for a change in spin multiplicity. We assigned the approximately 30 meV activation energy extracted from optical and trESR spectroscopy to ^4^[D_0_T_1_] → ^2^CT transfer. After the ^2^CT state is reformed, a hole transfer from anthracene onto the carbazole can rapidly yield the emissive ^2^[D_1_S_0_] state. At temperatures below 100 K, phosphorescence is observed. This can originate either from ^4^[D_0_T_1_] in a spin-forbidden process at the lowest temperatures or from ^2^[D_0_T_1_] in a spin-allowed process. The increase in phosphorescence intensity with temperature in this regime confirms that ^4^[D_0_T_1_] is the lowest energy excited state (that is, that the exchange is ferromagnetic).

The presence of a bridging phenyl in TTM-1Cz-PhAn led to a loss of PLQE because energy transfer (Supplementary Fig. 11), RISC and emission (Extended Data Fig. 3) are slow compared with TTM-1Cz-An. The observed PL red-shift could be due to a larger electron–hole separation in TTM-1Cz-PhAn, which leads to a less emissive state. Despite the greater spatial separation between the radical and anthracene, TTM-1Cz-PhAn remained in the strong exchange coupling regime (Extended Data Fig. 5).

The small energy offsets between excited states involved in this mechanism likely led to a high degree of mixing between states with overall doublet multiplicity (Fig. 3d), as indicated by calculated electronic couplings (Supplementary Table 12)^34^. We detected signatures of ^2^[D_1_S_0_] and ^2^[D_0_T_1_] mixing in both our ultrafast optical spectroscopy and low-temperature excitation scans. Although these effects were probably modulated by conformational reorganization, vibrational motion and environment dynamics, the quartet–doublet energy gap is always in the strong exchange regime as demonstrated by our ESR experiments. Therefore our system benefits from low energy offsets on the optical scales but large energy offsets on the magnetic scales. This enables access to robust, high-spin excited states coupled to an efficient emissive state.

## Optical readout at room temperature

**Extended Data Fig. 6:**
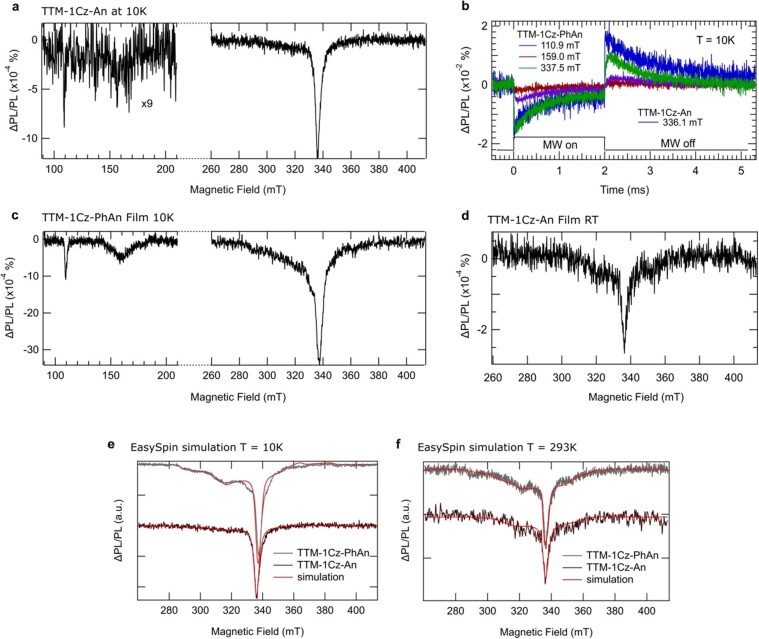
ODMR. **a**, cwODMR at 10K of dilute frozen toluene solution of TTM-1Cz-An. **b**, trODMR at 10 K for TTM-1Cz-PhAn HF and FF signals (purple, red, green) and TTM-1Cz-An FF signal (blue), revealing a negative sign for all signals. **c**, cwODMR spectra of 5% TTM-1Cz-PhAn in PMMA film at 10 K. **d**, cwODMR spectrum of 5% TTM-1Cz-An in PMMA film at 293 K, showing quartet contribution at room temperature. **e**, Simulations (red) for TTM-1Cz-PhAn (grey) and TTM-1Cz-An (black) at T = 10 K (left) and T = 293 K (right).

The luminescence of our materials opens the path toward optical readout in organic high-spin molecules, which we explored with optically detected magnetic resonance (ODMR) measurements. Dilute frozen toluene solutions of R-A at 10 K showed a broad, patterned full-field ODMR signal (Fig. 3e), matching quartet simulation parameters of trESR. As in trESR, we also observed half-field ODMR signals with Δ*m*_s_ = 3 and Δ*m*_s_ = 2 transitions at *g* = 6.17 and *g* = 4.23, respectively. To achieve readout at ambient temperatures we performed ODMR on dilute PMMA films of the two R-A molecules. Both TTM-1Cz-An and TTM-1Cz-PhAn films showed a clear ODMR contrast at room temperature (Fig. 3f and Extended Data Fig. 6). The resonant PL change in films at 295 K is comparable to that observed at 10 K. The dipolar parameters remained unchanged (Extended Data Table 1), confirming assignment to the quartet state. No ODMR signals were detected for TTM-1Cz, indicating identical emissive rates for *m*_s_ = ±1/2 D_1_ sublevels in an isolated radical. The ODMR contrast in R-A is thus due to the ^4^[D_0_T_1_] origin of ^2^[D_1_S_0_] emission and demonstrates optical readout of the quartet state at room temperature.

To investigate the sign of the ODMR signal we performed transient ODMR (trODMR) measurements, which directly probe PL change in resonant conditions using digitizer detection^35^. ODMR transients with application of 2 ms square microwave pulses at both full-field and half-field show a negative sign for all signals (Fig. 3e inset and Extended Data Fig. 6). Although at lower temperatures the quartet state is coupled to phosphorescence, at higher temperatures quartet depopulation occurs via RISC. The negative sign of the ODMR signal suggests that microwaves drive transitions from the more populated *m*_s_ = ±1/2 to less efficiently linked *m*_s_ = ±3/2 quartet sublevels, thus decreasing PL from the doublet state under resonant conditions.

**Fig. 4:**
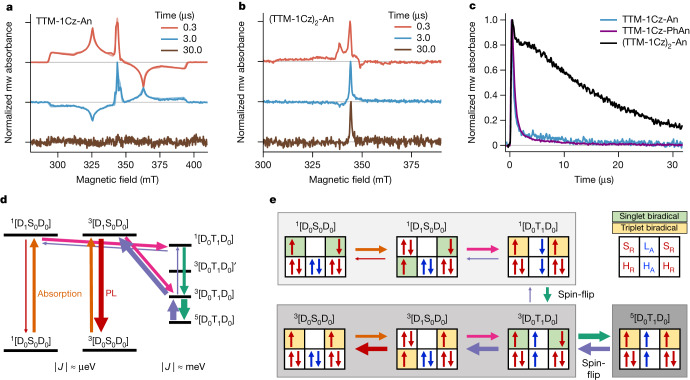
Room-temperature spin dynamics and ground-state control. **a**, X-band (9.7 GHz) trESR spectra on 5% PMMA films of TTM-1Cz-An under 600 nm excitation at 295 K (darker lines) and their simulations (lighter lines). Noise of 30 µs trace scaled to match noise level in **b**. **b**, TrESR spectra on 5% PMMA films of (TTM-1Cz)_2_-An under 532 nm excitation at 295 K, showing a long-lived *g* = 2.00 (344.5 mT) signal. **c**, Comparison of *g* = 2.00 kinetics in trESR on 5% PMMA films at 295 K. Long-lived doublet polarization was seen in R-A-R but not in R-A. **d**, (TTM-1Cz)_2_-An energy level diagram showing two exchange coupling regimes. In the ground and initially photogenerated states the radical spins were uncorrelated (|*J|* ≈ µeV) but became strongly coupled when the T_1_ wavefunction was present (|*J|* ≈ meV) **e**, Summary scheme of dominant orbital contributions during quintet formation. The triplet channel was kinetically more favoured during temperature-activated emission, which led to a strong polarization of the ground state.

**Extended Data Fig. 7:**
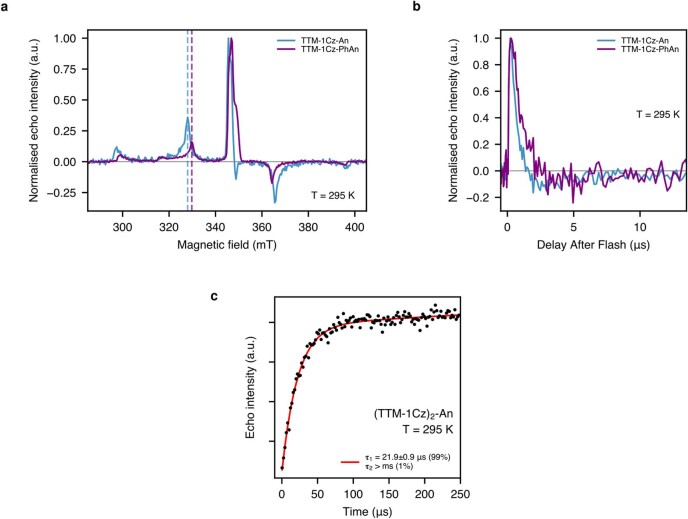
Room temperature pulsed ESR. **a**, Echo Detected Field Sweep (EDFS) of excited quartet states of R-A at 295 K in 5% radical in PMMA 0.5 μs after 1.4 mJ 600 nm excitation. **b**, Delay After Flash (DAF) scans at quartet field positions (marked with dashed vertical lines in panel a). **c**, Spin-lattice relaxation of R-A-R ground state. 5% (TTM-1Cz)_2_-An in PMMA film at 295 K. Experiment performed at field corresponding to *g* = 2.00355 at X-band (345.65 mT) without light excitation. Inversion recovery pulse sequence.

As in ODMR, we observed spin-polarized quartet signals in room-temperature trESR on PMMA films of R-A (Fig. 4a). We additionally performed room-temperature pulsed ESR experiments (Extended Data Fig. 7). Quartet echoes were detectable up to 10 μs following photoexcitation at 295 K, further demonstrating the potential of these molecules as optically addressable qudits in the solid state.

## Biradical ground-state control

The presence of two radical spins in luminescent R-A-R allows engineering of more complex ground and excited-state interactions. As shown above, the photophysical properties of the (TTM-1Cz)_2_-An biradical are analogous to TTM-1Cz-An, with ultrafast wavefunction localization onto the anthracene after light absorption followed by delayed emission. However, the biradical nature of R-A-R has significant consequences for spin properties compared with R-A monoradicals. In the R-A-R ground state the exchange interaction between the two D_0_ electrons is extremely weak (micro-electron volts or lower), as shown by the exclusively doublet transient nutation signals in the dark and temperature dependence of continuous-wave ESR intensity (Supplementary Fig. 19). This is consistent with the large spin–spin distance of 2.09 ± 0.19 nm determined by double-electron–electron resonance spectroscopy (Supplementary Fig. 20). Therefore, the biradical spin pair is uncorrelated in the ground state and the ^1^[D_0_S_0_D_0_] and ^3^[D_0_S_0_D_0_] states are degenerate, as in a true biradical^36^.

Coupling between D_0_ electrons can be transiently switched on due to strong (around milli-electron volts) exchange within [D_0_T_1_D_0_] excited states, and we detected polarized ^5^[D_0_T_1_D_0_] quintet (trip-quintet) in room-temperature trESR (Fig. 4b). The absence of ^3^[D_0_T_1_D_0_] triplet (‘trip-triplet’) signals confirms that the quintet is the lowest excited state and is generated efficiently. Following relaxation back to the ground state, the spin information of D_0_ biradical electrons is preserved. This is evidenced by the long-lived *g* = 2.00 polarization extending beyond 30 µs at room temperature (Fig. 4c), well past any luminescence or quintet polarization. Such ground-state polarization is absent in R-A monoradicals.

To understand its origin we examined the energy level structure of R-A-R. Because singlet and triplet ground states are degenerate, both contribute to the forward process of quintet state generation (Fig. 4d). The singlet and triplet channels each provide an efficient, rapid, spin-conserving pathway to a [D_0_T_1_D_0_] state analogous to the doublet pathway in R-A. However, because quintet depopulation involves one spin-flip to access ^3^[D_0_T_1_D_0_] but two spin-flips to reach ^1^[D_0_T_1_D_0_], the triplet channel dominates the reverse process (Fig. 4e). Because the ^3^[D_1_S_0_D_0_] state can then quickly form and decay radiatively, this leads to an excess of ^3^[D_0_S_0_D_0_] ground state. Despite ground-state energetic degeneracy, this method allows for a preferential preparation of the triplet biradical configuration. Only following light-induced quintet generation and relaxation, the ground-state biradical consists of a spin-polarized pair of electrons that lose spin alignment with a long lifetime of 20 ± 1 μs at room temperature in solid films. This timescale matches the spin-lattice relaxation time of D_0_ electrons measured in pulsed ESR on the same (TTM-1Cz)_2_-An film at 295 K (Extended Data Fig. 7), and could be extended further by host engineering and deuteration.

## Conclusions

Through our study combining optical spectroscopy, ESR, ODMR and theoretical modelling we have demonstrated that we can generate pure high-spin states in organic molecules, manipulate them and then read them optically (Fig. 3c). Using a luminescent radical and engineering an excited-state manifold with small energy offsets, we have shown a new pathway to generate high-spin states, here exemplified as a quartet or quintet. The radical can dress the triplet exciton in hybridized states that can reversibly access the high-spin manifold. Consequently, a luminescent state can be restored from addressable high-spin states. Remarkably, we observed polarized high-spin states via ESR and ODMR at room temperature in non-crystalline solid state, showing real potential for future applications such as quantum sensing.

Our approach of coherent manipulation of high-spin states coupled with emission complements insights gained from alternative qubit platforms such as colour centres—in particular, the diamond nitrogen vacancies in which optical spin polarization is also generated by excited-state intersystem crossing. Whereas nitrogen vacancy-negative centres show long spin coherence times at room temperature, their scaleability may be limited by the challenges of controlling defect placement and preventing decoherence in non-isolated defects^37^. By contrast, molecules offer unparalleled chemical tunability due to a wealth of synthetic approaches and the potential to develop more extended spin structures, as demonstrated here by the behaviour of R-A and R-A-R. The scope for both chemical tuning and extension to polyradical structures opens new opportunities for designs of coupled spin systems that can be addressed with light, noting that their placement and intermolecular interactions can be controlled with self-assembly and scanning probe methods.

## Methods

### Transient absorption spectroscopy

Transient absorption experiments were conducted on a setup pumped by a regenerative Ti:sapphire amplifier (Solstice Ace, Spectra-Physics) emitting 100-fs pulses centred at 800 nm at a rate of 1 kHz and a total output of 7 W. Depending on the probed spectral range and timescales, different combinations of optical systems were used.

To collect sub-nanosecond dynamics in the visible range, frequency-doubled output of the amplifier was used to seed a home-built broadband non-collinear optical parametric amplifier (NOPA) tuned to output 530–750 nm pulses with a beta barium borate mixing crystal (Eksma Optics). Alternatively, to probe the infrared range the output of the amplifier was used to seed a home-built NOPA tuned to output 1,250–1,700 nm pulses with a periodically poled stoichiometric lithium tantalate mixing crystal. Following chirp-correction the white light output was split on a 50/50 beam splitter, focused to below 200 μm and used as the probe and reference beams. Wavelength-tuneable pump pulses were generated in a home-built visible narrowband NOPA. The pump and probe beams were spatially overlapped at the focal point using a beam profiler, with the pump spot diameter at least fivefold larger than the probe. Time resolution was achieved by the introduction of a stepped optical delay (Thorlabs DDS300-E/M) between pump and probe pulses, with a computer-controlled delay stage allowing for maximum delay of 1.9 ns and beam wander of the probe due to changing beam pointing minimized to below 5 μm using a beam profiler. Pump pulses were chopped at 500 Hz to enable shot-to-shot referencing, which accounted for intensity fluctuations in the amplifier. After passing through the sample, the probe and reference beams were dispersed with a grating spectrometer (Shamrock SR303i, Andor Technology) and simultaneously measured with charge-coupled device (CCD) detector arrays (Entwicklungsbüro Stresing).

To collect sub-nanosecond dynamics in the ultraviolet range, the output of the amplifier was used to seed a home-built broadband NOPA tuned to output 350–650 nm pulses generated by focusing the 800 nm fundamental beam onto a CaF_2_ crystal (Eksma Optics, 5 mm) connected to a digital motion controller (Mercury C-863 DC Motor Controller), after passing through a mechanical delay stage. The transmitted pulses were collected with a single-line scan camera (JAI SW-4000M-PMCL) after passing through a spectrograph (Andor Shamrock SR-163).

### Transient PL spectroscopy

Time-resolved PL spectra were collected using an electrically gated intensified CCD (ICCD) camera (Andor iStar DH740 CCI-010) coupled with an image identifier tube after passing through a calibrated grating spectrometer (Andor SR303i). The spectrometer input slit width was 200 μm. Samples were excited using pump pulses obtained from a home-built narrowband NOPA driven by the same amplifier as the transient optical absorption setups. A suitable long-pass filter was placed directly in front of the spectrometer to avoid scattered laser signals entering the camera. The kinetics were obtained by setting the gate delay steps with respect to the excitation pulse. The gate widths of the ICCD were 5 ns, 50 ns, 500 ns, 5 μs and 50 μs, with overlapping time regions used to compose decays.

Temperature-dependent measurements were performed using a closed-circuit pressurized helium cryostat (Optistat Dry BL4, Oxford Instruments), a compressor (HC-4E2, Sumitomo) and a temperature controller (Mercury iTC, Oxford Instruments). The vacuum level inside the cryostat was below 10^−5^ mbar.

### ESR

X-band ESR was acquired with either a Bruker Biospin E680 or E580 EleXSys spectrometer using a Bruker ER4118-MD5-W1 dielectric TE01_δ_ mode resonator (around 9.70 GHz) in an Oxford Instruments CF935 cryostat. Q-band ESR employed an ER5106QT-2w resonator and a conventional 1.5 T electromagnet, as for X-band frequencies. The amplifiers for pulsed ESR (Applied Systems Engineering) had saturated powers of 1.5 kW at the X-band and 180 W at the Q-band. Temperature was maintained with an ITC-503S temperature controller and a CF-935SW helium flow cryostat (both Oxford Instruments).

For laser-induced transient signals, photoexcitation was provided by a tunable Ekspla NT230 operating at a repetition rate of 50 Hz. Laser pulse energies used were 0.5 – 1.0 mJ, with pulse lengths of 3 ns transmitted at roughly 40% through the cryostat, microwave shield and resonator windows. A liquid-crystal depolarizer (DPP-25, ThorLabs) was placed in the laser path for all measurements unless otherwise indicated. Triggering of the LASER and ESR spectrometer involved synchronization with a Stanford Research Systems delay generator, DG645. Quadrature mixer detection was used in pulsed- and continuous-wave detection.

Transient continuous-wave ESR spectra were simulated using EasySpin (Supplementary Information Section 2b)^38^. To account for the effective deviation from isotropic ordering due to magnetophotoselection effects^39^ we introduced an ordering term of the form

$$I({\rm{\phi }},{\rm{\theta }})={\rm{\exp }}(0.5\times {O}_{{\rm{\theta }}}\times (3\times {{\rm{\cos }}}^{2}({\rm{\theta }})-1)+{O}_{{\rm{\phi }}}\times ({{\rm{\sin }}}^{2}({\rm{\theta }}){\rm{\cos }}(2{\rm{\phi }}))),$$

where *O*_θ_ and *O*_ϕ_ are θ and ϕ angle ordering parameters, respectively; *O*_θ_ was set to zero in all simulations.

### ODMR

Optically detected magnetic resonance experiments were carried out using a modified X-band spectrometer (Bruker E300) equipped with a continuous-flow helium cryostat (Oxford ESR 900) and a microwave cavity (Bruker ER4104OR, approximately 9.43 GHz) with optical access. Optical irradiation was performed with a 532 nm continuous-wave laser (Cobolt Samba CW 532 nm DPSSL) from one side-opening of the cavity. PL was detected with a silicon photodiode (Hamamatsu S2281) on the opposite opening, using a 561 nm long-pass filter to reject excitation light. The PL signal was amplified by a current/voltage amplifier (Femto DHPCA-100). For continuous-wave ODMR, PL was recorded by a lock-in detector (Ametek SR 7230) referenced by on–off modulation of microwaves with a frequency of 547 Hz. Microwaves were generated with a microwave signal generator (Anritsu MG3694C), amplified to 3 W (Microsemi) and guided into the cavity. For trODMR, PL was recorded by a digitizer card (GaGe Razor Express 1642 CompuScope) whereby a pulse blaster card (PulseBlasterESR-PRO) triggered the digitizer card and produced microwave pulses for a set length. Microwaves were generated with the same microwave signal generator as in continuous-wave ODMR, whereby they were amplified to 5 W by a travelling wave tube amplifier (Varian VZX 6981 K1ACDK) and guided into the cavity.

### Theoretical calculations

The doublet ground-state ^2^[D_0_S_0_] and quartet ^4^[D_0_T_1_] of R-A monoradicals TTM-1Cz-An and TTM-1Cz-PhAn were optimized by means of unrestricted Kohn–Sham formalism within the density functional theory framework, using the ωB97X-D exchange-correlation functional and the 6–31G(d,p) basis set. In both ^2^[D_0_S_0_] and ^4^[D_0_T_1_] optimized structures, spin contamination was predicted to be negligible (less than 5%). The R-A-R biradical (TTM-1Cz)_2_-An ground-state ^3^[D_0_S_0_D_0_] was optimized with the same level of theory as described above. Broken-symmetry density functional theory calculations pointed to a degeneracy between the triplet ^3^[D_0_S_0_D_0_] and the broken-symmetry singlet ground-state ^1^[D_0_S_0_D_0_], which was found to lie less than 0.05 cm^−1^ above the ^3^[D_0_S_0_D_0_] configuration. These calculations were performed with Gaussian16 software^40^.

To gain access to all relevant configuration state functions of R-A, state-averaged complete active-space, self-consistent field (CASSCF) calculations were performed on the optimized monoradical TTM-1Cz-An ground-state ^2^[D_0_S_0_] structure using the Def2-TZVP basis set^41^. On top of a converged CASSCF wave function, strongly contracted second-order N-electron valence state perturbation theory (NEVPT2) calculations were performed to recover the missing dynamic electronic correlation at the CASSCF level^42^. Because the CASSCF wave function is expanded in terms of Slater determinants computed in a restricted-open-shell formalism, both CASSCF and NEVPT2 methods provide excited states free from spin contamination. The same computational methods were applied to the optimized biradical (TTM-1Cz)_2_-An ground-state ^3^[D_0_S_0_D_0_] structure, using a smaller basis set (Def2-SVP) to reduce computational costs. Such calculations were run with ORCA 4.2 code^43^.

Electronic couplings between the diabatic doublet states ^2^[D_0_T_1_], ^2^[D_1_S_0_] and ^2^CT of the monoradical TTM-1Cz-An were estimated via a diabatization procedure considering the Boys localization scheme, where adiabatic states were computed with the restricted active-space configuration interaction (RAS–CI) method^44^, along with the Def2-SVP basis set within Q-Chem 5.4 software^45^. In RAS–CI formalism the molecular orbital space is divided into three subspaces, RAS1, RAS2 and RAS3. The excited configurations are generated by an excitation operator ($\hat{R}$) acting on the restricted-open-shell Hartree–Fock reference wave function (*ϕ*_0_):

$$| {\varPsi }^{{\rm{RAS-CI}}}\rangle =\hat{R}| {\phi }_{0}\rangle $$

In the current RAS–CI implementation of Q-Chem 5.4, $\hat{R}$ is defined as

$$\hat{R}={\hat{r}}^{{\rm{RAS}}2}+{\hat{r}}^{{\rm{hole}}}+{\hat{r}}^{{\rm{particles}}}$$

where ${\hat{r}}^{{\rm{RAS}}2}$ generates all possible electronic configurations (singles, doubles, triples and so on) in the RAS2 subspace, corresponding to full CI treatment within the selected subspace; ${\hat{r}}^{{\rm{hole}}}$ generates electronic configurations by promotion of single excitations from RAS1 to RAS2, creating *n* holes in the RAS1 subspace; analogously, ${\hat{r}}^{{\rm{particles}}}$ generates electronic configurations by promotion of electrons from RAS2 to RAS3, thus creating *m* particles in RAS3. In our case RAS2 is built from 11 electrons in ten orbitals, allowing recovery of all relevant states (that is, ^2^[D_1_S_0_], ^2/4^[D_0_T_1_] and ^2^CT) predicted at the NEVPT2 level; RAS1 (RAS3) facilitates the creation of six holes (particles) whereas the remaining MOs remain doubly occupied (unoccupied). Like CASSCF and NEVPT2, the RAS–CI approach allows excited states free from spin contamination.

In the Boys localization scheme, the diabatic states are written as a linear combination of adiabatic states:

$$| {\Xi }_{\varGamma }\rangle =\mathop{\sum }\limits_{J}^{{N}_{{\rm{Adiab}}}}| {\varPsi }_{J}\rangle {U}_{J\varGamma }$$

with $| {\Xi }_{\varGamma }\rangle$ the *Γ*th diabatic state, *N*_Adiab_ the number of adiabatic states, $| {\varPsi }_{J}\rangle$ the *J*th adiabatic state and ${U}_{J\varGamma }$ the element of the rotation matrix from the adiabatic to the diabatic representation. With such a scheme, the electric dipole moment difference between each pair of diabatic states is maximized:

$${f}_{{\rm{Boys}}}\left(U\right)={f}_{{\rm{Boys}}}\left(\left\{\varXi \right\}\right){\boldsymbol{=}}\mathop{\sum }\limits_{\varGamma ,\varDelta =1}^{{N}_{{\rm{Adiab}}}}{\left|\left\langle {\varXi }_{\varGamma },| ,\hat{\mu },| ,{\varXi }_{\varGamma }\right\rangle -\left\langle {\varXi }_{\varDelta },| ,\hat{\mu },| ,{\varXi }_{\varDelta }\right\rangle \right|}^{2}$$

As a result we obtain the rotation matrix *U*, which transforms the Hamiltonian from a (diagonal) adiabatic to a (non-diagonal) diabatic representation. The matrix elements of the diabatic Hamiltonian $\left\langle {\varXi }_{\varGamma },| ,\hat{{\rm{H}}},| ,{\varXi }_{\triangle }\right\rangle$ represent either the diabatic state energy when *Γ* = *∆* or the electronic coupling between diabatic states when *Γ* ≠ *∆*. Diabatization was carried out on top of RAS–CI excited states by systematically increasing its size—that is, increasing the number of adiabatic states. In 3 × 3 diabatization only the adiabatic states associated with ^2^[D_1_S_0_], ^2^[D_0_T_1_] and ^2^CT were considered, whereas for 9 × 9 and 17 × 17 the first nine and 17 adiabatic excited states were introduced, respectively.

## Online content

Any methods, additional references, Nature Portfolio reporting summaries, source data, extended data, supplementary information, acknowledgements, peer review information; details of author contributions and competing interests; and statements of data and code availability are available at 10.1038/s41586-023-06222-1.

### Supplementary information

Supplementary Information This file contains Supplementary Sections 1–3. 1, Synthesis and characterization, including Supplementary Figs. 1–10. 2, Supplementary experimental results, including Supplementary Figs. 11–20. 3, Quantum chemical calculations, including Supplementary Figs. 21–31, Tables 1–17 and References.

## Figures and Tables

**Extended Data Table 1:**
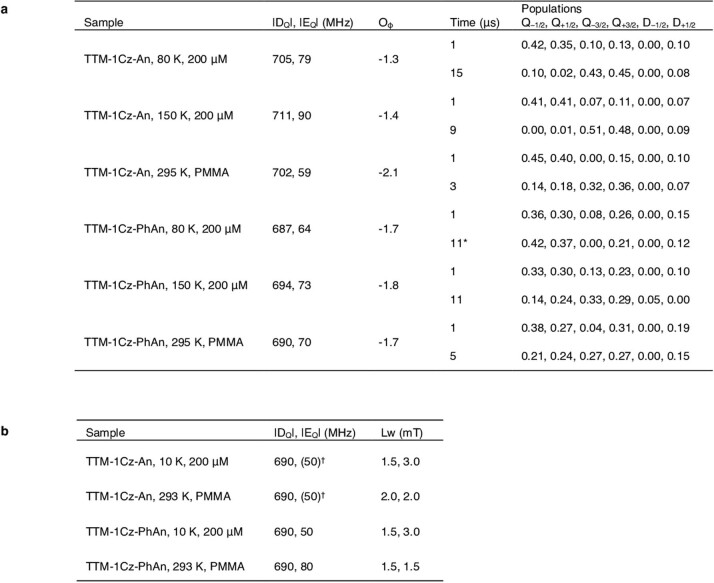
R-A Spin Hamiltonian modelling

**a**, Simulation parameters for full-field X-band trESR spectra. g_T_ = 2.003, g_R_ = 2.00355. Linewidth was set to 1.5 mT in all simulations. Populations at maximum absolute polarisation times before and after inversion. *: Not inverted within acquisition timescale. **b**, Simulation parameters for ODMR spectra. g_T_ = 2.003, g_R_ = 2.003, O_ɸ_ = 0. Linewidth (Lw) given as Gaussian, Lorentzian. ^†^: E value cannot be determined as the X and Y transitions are weakly pronounced.

## Data Availability

The data underlying all figures in the main text are publicly available from the University of Cambridge repository: 10.17863/CAM.96533.

## Code availability

All code used in this work is available from the corresponding authors on reasonable request.

## References

[1] Wasielewski MR (2020). Exploiting chemistry and molecular systems for quantum information science. Nat. Rev. Chem..

[2] Atzori M, Sessoli R (2019). The second quantum revolution: role and challenges of molecular chemistry. J. Am. Chem. Soc..

[3] Yu C-J, von Kugelgen S, Laorenza DW, Freedman DE (2021). A molecular approach to quantum sensing. ACS Cent. Sci..

[4] Gaita-Ariño A, Luis F, Hill S, Coronado E (2019). Molecular spins for quantum computation. Nat. Chem..

[5] Awschalom DD, Hanson R, Wrachtrup J, Zhou BB (2018). Quantum technologies with optically interfaced solid-state spins. Nat. Photonics.

[6] Quintes T, Mayländer M, Richert S (2023). Properties and applications of photoexcited chromophore–radical systems. Nat. Rev. Chem..

[7] Teki, Y. Excited-state dynamics of non-luminescent and luminescent π-radicals. *Chemistry* **26**, 980–996 (2020). 10.1002/chem.201903444 31479154

[8] DiVincenzo DP (2000). The physical implementation of quantum computation. Fortschr. Phys..

[9] Bayliss SL (2020). Optically addressable molecular spins for quantum information processing. Science.

[10] Bader K (2014). Room temperature quantum coherence in a potential molecular qubit. Nat. Commun..

[11] Warner M (2013). Potential for spin-based information processing in a thin-film molecular semiconductor. Nature.

[12] Wang D (2019). Turning a molecule into a coherent two-level quantum system. Nat. Phys..

[13] Filidou V (2012). Ultrafast entangling gates between nuclear spins using photoexcited triplet states. Nat. Phys..

[14] Oxborrow M, Breeze JD, Alford NM (2012). Room-temperature solid-state maser. Nature.

[15] Giacobbe EM (2009). Ultrafast intersystem crossing and spin dynamics of photoexcited perylene-3,4:9,10-bis(dicarboximide) covalently linked to a nitroxide radical at fixed distances. J. Am. Chem. Soc..

[16] Kollmar C, Sixl H (1982). Theory of a coupled doublet-triplet system. Mol. Phys..

[17] Teki Y, Miyamoto S, Nakatsuji M, Miura Y (2001). π-Topology and spin alignment utilizing the excited molecular field: observation of the excited high-spin quartet (S = 3/2) and quintet (S = 2) states on purely organic π-conjugated spin systems. J. Am. Chem. Soc..

[18] Leuenberger MN, Loss D (2001). Quantum computing in molecular magnets. Nature.

[19] Moreno-Pineda E, Godfrin C, Balestro F, Wernsdorfer W, Ruben M (2018). Molecular spin qudits for quantum algorithms. Chem. Soc. Rev..

[20] Mayländer M, Chen S, Lorenzo ER, Wasielewski MR, Richert S (2021). Exploring photogenerated molecular quartet states as spin qubits and qudits. J. Am. Chem. Soc..

[21] Rozenshtein V (2005). Electron spin polarization of functionalized fullerenes. Reversed quartet mechanism. J. Phys. Chem. A.

[22] Peng, Q., Obolda, A., Zhang, M. & Li, F. Organic light-emitting diodes using a neutral π radical as emitter: the emission from a doublet. *Angew. Chem. Int. Ed. Engl.* **54**, 7091–7095 (2015). 10.1002/anie.201500242 25916621

[23] Matsuoka R, Mizuno A, Mibu T, Kusamoto T (2022). Luminescence of doublet molecular systems. Coord. Chem. Rev..

[24] Li X, Wang Y-L, Chen C, Ren Y-Y, Han Y-F (2022). A platform for blue-luminescent carbon-centered radicals. Nat. Commun..

[25] Ai X (2018). Efficient radical-based light-emitting diodes with doublet emission. Nature.

[26] Gamero V (2006). [4-(N-Carbazolyl)-2,6-dichlorophenyl]bis(2,4,6-trichlorophenyl)methyl radical an efficient red light-emitting paramagnetic molecule. Tetrahedron Lett..

[27] Khan ZH (1992). Electronic spectra of radical cations and their correlation with photoelectron spectra. VI. A reinvestigation of two-, three-, and four-ring condensed aromatics. Acta Phys. Pol. A.

[28] Padhye MR, Mcglynn SP, Kasha M (1956). Lowest triplet state of anthracene. J. Chem. Phys..

[29] Biskup T (2019). Structure-function relationship of organic semiconductors: detailed insights from time-resolved EPR spectroscopy. Front. Chem..

[30] Köhler SD, Höfel S, Drescher M (2013). Triplet state kinetics of anthracene studied by pulsed electron paramagnetic resonance. Mol. Phys..

[31] Mizuochi N, Ohba Y, Yamauchi S (1997). A two-dimensional EPR nutation study on excited multiplet states of fullerene linked to a nitroxide radical. J. Phys. Chem. A.

[32] Moreno-Pineda, E., Martins, D. O. T. A. & Tuna, F. Molecules as qubits, qudits and quantum gates. In *Electron Paramagnetic Resonance* Vol. 27 (eds Chechik, V., Murphy, D. M. & Bode, B. E.) 146–187 (Royal Society of Chemistry, 2021).

[33] El-Sayed MA (1968). Triplet state. Its radiative and nonradiative properties. Acc. Chem. Res..

[34] Chen M (2018). Singlet fission in covalent terrylenediimide dimers: probing the nature of the multiexciton state using femtosecond mid-infrared spectroscopy. J. Am. Chem. Soc..

[35] Grüne J, Dyakonov V, Sperlich A (2021). Detecting triplet states in opto-electronic and photovoltaic materials and devices by transient optically detected magnetic resonance. Mater. Horiz..

[36] Abe M (2013). Diradicals. Chem. Rev..

[37] Wozfowicz G (2021). Quantum guidelines for solid-state spin defects. Nat. Rev. Mater..

[38] Stoll S, Schweiger A (2006). EasySpin, a comprehensive software package for spectral simulation and analysis in EPR. J. Magn. Reson..

[39] Tait CE, Neuhaus P, Anderson HL, Timmel CR (2015). Triplet state delocalization in a conjugated porphyrin dimer probed by transient electron paramagnetic resonance techniques. J. Am. Chem. Soc..

[40] Frisch, M. J. et al. Gaussian 16, Revision C.01 (2016).

[41] Roos, B. O. The complete active space self-consistent field method and its applications in electronic structure calculations. In *Advances in Chemical Physics* (ed. Lawley, K. P.) 399–445 (John Wiley & Sons, 1987).

[42] Angeli C, Cimiraglia R, Malrieu J-P (2001). N-electron valence state perturbation theory: a fast implementation of the strongly contracted variant. Chem. Phys. Lett..

[43] Neese F, Wennmohs F, Becker U, Riplinger C (2020). The ORCA quantum chemistry program package. J. Chem. Phys..

[44] Casanova D, Head-Gordon M (2009). Restricted active space spin-flip configuration interaction approach: theory, implementation and examples. Phys. Chem. Chem. Phys..

[45] Epifanovsky E (2021). Software for the frontiers of quantum chemistry: an overview of developments in the Q-Chem 5 package. J. Chem. Phys..

