# Supplementary material for: Reversible spin-optical interface in luminescent organic radicals
Source: Nature. 2023 Aug 16;620(7974):538–44. doi: 10.1038/s41586-023-06222-1 (PMC10432275; doi:10.1038/s41586-023-06222-1)
Supplement: Supplementary file 1 — This file contains Supplementary Sections 1–3. 1, Synthesis and characterization, including Supplementary Figs. 1–10. 2, Supplementary experimental results, including Supplementary Figs. 11–20. 3, Quantum chemical calculations, including Supplementary Figs. 21–31, Tables 1–17 and References. [file 41586_2023_6222_MOESM1_ESM.pdf]

nature portfolio

https://doi.org/10.1038/s41586-023-06222-1

Supplementary information

Reversible spin-optical interface in luminescent organic radicals

In the format provided by the authors and unedited

Supplementary Materials for

Reversible spin-optical interface in luminescent organic radicals

Sebastian Gorgon[1,2,*], Kuo Lv[3], Jeannine Grüne[4,†], Bluebell H. Drummond[1], William K. Myers[2], Giacomo Londi[5], Gaetano Ricci[5], Danillo Valverde[5], Claire Tonnelé[6], Petri Murto[7], Alexander S. Romanov[8], David Casanova[6], Vladimir Dyakonov[4], Andreas Sperlich[4], David Beljonne[9], Yoann Olivier[5], Feng Li[3], Richard H. Friend[1,*], Emrys W. Evans[10,*]

[1] Cavendish Laboratory, University of Cambridge; JJ Thomson Ave, Cambridge, CB3 0HE, United Kingdom
[2] Centre for Advanced Electron Spin Resonance, Department of Chemistry, University of Oxford; Inorganic Chemistry Laboratory, S Parks Rd, Oxford, OX1 3QR, United Kingdom
[3] State Key Laboratory of Supramolecular Structure and Materials, College of Chemistry, Jilin University; Qianjin Avenue 2699, Changchun, 130012, P. R. China
[4] Experimental Physics VI, Faculty of Physics and Astronomy, University of Würzburg; 97074 Würzburg, Germany
[5] Laboratory for Computational Modelling of Functional Materials, Namur Institute of Structured Matter, University of Namur; Rue de Bruxelles 61, 5000 Namur, Belgium
[6] Donostia International Physics Centre; Donostia, Euskadi, Spain
[7] Yusuf Hamied Department of Chemistry, University of Cambridge; Cambridge, CB2 1EW, United Kingdom
[8] Department of Chemistry, University of Manchester; Manchester, M13 9PL, United Kingdom
[9] Laboratory for Chemistry of Novel Materials, University of Mons; Place du Parc 20, 7000 Mons, Belgium
[10] Department of Chemistry, Swansea University; Singleton Park, Swansea, SA2 8PP, United Kingdom
[†] Present address: Cavendish Laboratory, University of Cambridge; JJ Thomson Ave, Cambridge, CB3 0HE, United Kingdom

*Corresponding authors. Email addresses: sg911@cam.ac.uk (S.G.), rhf10@cam.ac.uk (R.H.F.), emrys.evans@swansea.ac.uk (E.W.E.).

Sections:

1. Synthesis and characterisation
2. Supplementary experimental results
3. Quantum chemical calculations

1. Synthesis and characterisation

All chemical reagents and solvents were purchased from commercial suppliers and used without further purification (unless otherwise stated). Tetrahydrofuran (THF) was distilled before used. Column chromatography was performed with silica gel (200-300 mesh).

The ^{1}H nuclear magnetic resonance (NMR) spectra were recorded with a Bruker AVANCE-III 500 NMR spectrometer for samples in deuterated Dimethyl sulfoxide (DMSO) at ambient temperature. GC-MS mass spectra were measured using a Thermo Fisher ITQ1100 mass spectrometer. MALDI-TOF mass spectra were recorded on a Bruker Autoflex Speed TOF/TOF mass spectrometer with DCTB matrix.

Elemental analysis (EA) was performed on a Elementar Vario micro cube elemental analyzer.

Spin densities of target radicals 3,4 and 6 were estimated using ESR with an internal standard. The cw X-band spectra obtained in the dark in toluene solutions at room temperature were doubly integrated after baseline correction. This was subjected to a quantitative EPR analysis, considering the sample volume, cavity Q and resonator geometry. We estimate a 20% error in nominal concentrations due to the low masses of solids employed in preparation of the solutions for characterisation.

HTTM and TTM were prepared according to our previous report (Scheme 1).[22] A general procedure for the synthesis of radicals 3-4 is given in Scheme 2.

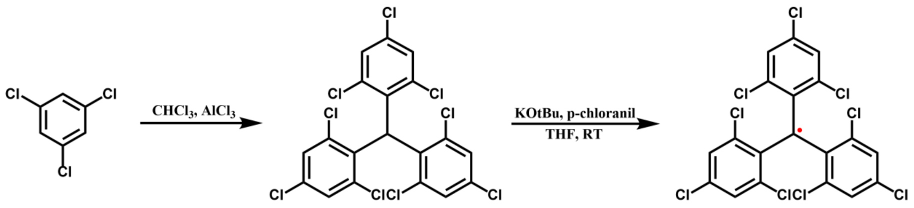

Scheme 1. Synthesis of HTTM and TTM.

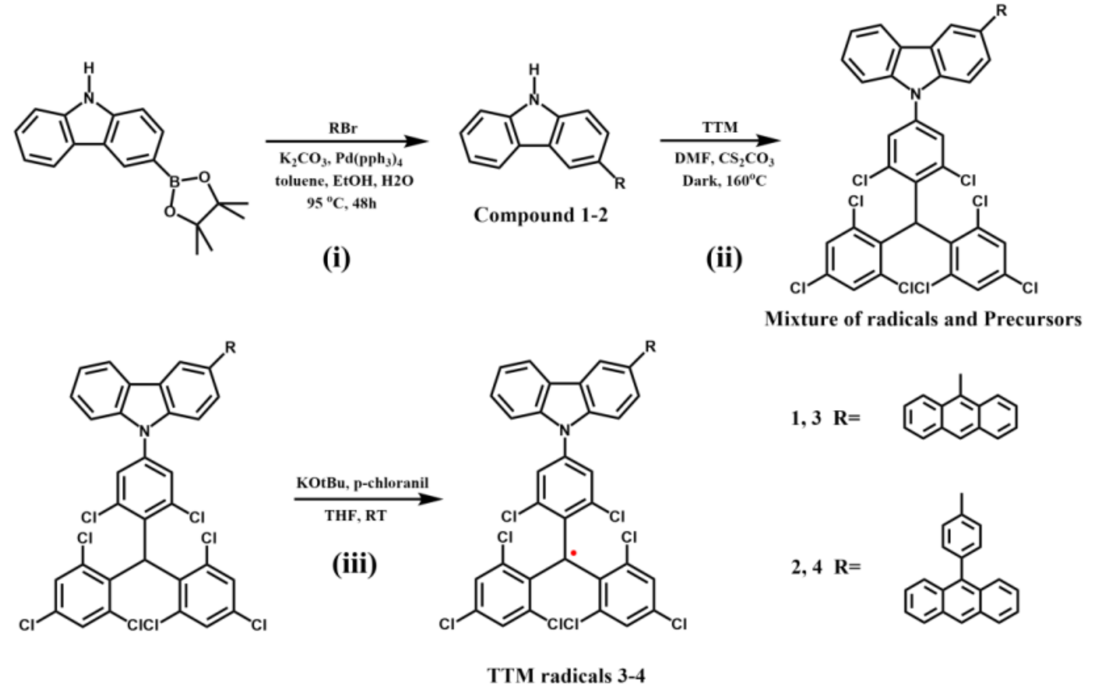

Scheme 2. Synthesis of TTM-1Cz-An (radical 3) and radical TTM-1Cz-PhAn (radical 4).

Scheme 2. (i): 3-(4,4,5,5-Tetramethyl-1,3,2-dioxaborolan-2-yl)carbazole (3.0 mmol) and 9-bromoanthracene (3.0 mmol) were dissolved in a mixed solvent of toluene (12 mL), K_2CO_3 aqueous solution (8 mL, 2 M) and ethanol (4 mL), then catalyst $Pd(PPh_3)_4$ (0.15 mmol) was added under argon atmosphere. The mixture was stirred at 95°C for 48 h under argon atmosphere and in the dark. Following this, the reaction mixture was cool to room temperature, and 5% hydrochloric acid was dropped plus until no bubbles were generated. After extraction with dichloromethane, the organic layer was collected and dried with anhydrous $MgSO_4$. The solvent was removed under vacuum and the crude product was purified by silica gel column chromatography (using petroleum ether: Ethyl acetate = 5:2, v/v). Desired compound 1 was obtained as white solids.

Compound 1：white powder(0.58 g, 56%)；^{1}H NMR (500 MHz, DMSO) δ 11.48 (s, 1H), 8.68 (s, 1H), 8.19 – 8.14 (m, 3H), 8.13 (d, J = 7.7 Hz, 1H), 7.72 (d, J = 8.1 Hz, 1H), 7.63 (d, J = 8.9 Hz, 2H), 7.57 (d, J = 8.2 Hz, 1H), 7.55 – 7.49 (m, 2H), 7.45 – 7.36 (m, 4H), 7.15 (t, J = 7.4 Hz, 1H). MS (GC-MS) (m/z):343.29 (Fig. S1).

Compound 2：white powder(0.72 g, 57%)；^{1}H NMR (500 MHz, DMSO) δ 11.37 (s, 1H), 8.71 (s, 1H), 8.64 (s, 1H), 8.27 (d, J = 7.8 Hz, 1H), 8.18 (d, J = 8.4 Hz, 2H), 8.04 (d, J = 8.0 Hz, 2H), 7.89 (d, J = 8.4 Hz, 1H), 7.69 (d, J = 8.8 Hz, 2H), 7.64 (d, J = 8.4 Hz, 1H), 7.57 – 7.51 (m, 5H), 7.49 – 7.40 (m, 3H), 7.21 (t, J = 7.4 Hz, 1H).
MS (GC-MS) (m/z):419.34 (Fig. S2).

Scheme 2. (ii): TTM (3.0 mmol), compound 1 (2.0 mmol), anhydrous Cs_2CO_3 (3.0 mmol) were added in DMF (20 ml). The mixture was stirred at 160°C for 12 h under argon atmosphere and dark conditions. After the reaction mixture was cooled to room temperatures and poured into (0.2

M) hydrochloric acid solution; the precipitate was filtered and washed with water three times. The crude product was dissolved in dichloromethane and extracted with water and dichloromethane. The organic layer was dried over $MgSO_4$ and evaporated under vacuum. The crude product was purified by silica gel column chromatography (using petroleum ether: dichloromethane = 9:1, v/v). Mixtures of the desired radical 3 and its precursor compounds were obtained.

Radical 3 and its precursor: (0.49 g, 28%)
Radical 4 and its precursor: (0.70 g, 35%)

Scheme 2. (iii): Under argon atmosphere and dark conditions, the KOtBu (1.5 mmol.) was added to the THF solution of radical 3 and its precursor mixtures (0.5 mmol.); The solution was stirred for 5 h at room temperature. Then tetrachloro-p-benzoquinone (2.5 mmol.) was added and the solution was stirred for another 2 h. After the reaction finished, the solvent was removed under vacuum, and the crude product was purified by silica gel column chromatography (using petroleum ether: dichloromethane = 9:1, v/v). The desired radical 3 was obtained. The synthesis of radical 4 follows the same route as that of radical 3.

Radical 3 = TTM-1Cz-An: (0.37 g, 85%)
MS (GC-MS) (m/z):859.75 (Fig. S3).
ESI-FTMS$^+$ Calcd. for $[C_{45}H_{22}Cl_8N]^+$: 855.9255. Found: *m/z* = 855.9270. (Fig. S7).
EA: Calcd. for $C_{45}H_{22}Cl_8N$: C 62.83, H 2.58, N 1.63. Found: C 63.00, H 2.99, N 1.67.
ESR Spin density: 64 μM in nominal 50±10 μM solution (1.3±0.3 ≈ 1 paramagnetic centre per molecule).

Radical 4 = TTM-1Cz-PhAn: (0.41 g, 88%)
MS (GC-MS) (m/z):935.43 (Fig. S4).
TOF-MS-ASAP$^+$ Calcd. for $[C_{51}H_{27}Cl_8N]^+$: 932.9652. Found: *m/z* = 932.9650. (Fig. S8).
EA: Calcd. for $C_{51}H_{27}Cl_8N$: C 65.42, H 2.80, N 1.50. Found: C 65.84, H 2.97, N 1.52.
ESR Spin density: 331 μM in nominal 300±60 μM solution (1.1±0.2 ≈ 1 paramagnetic centre per molecule).

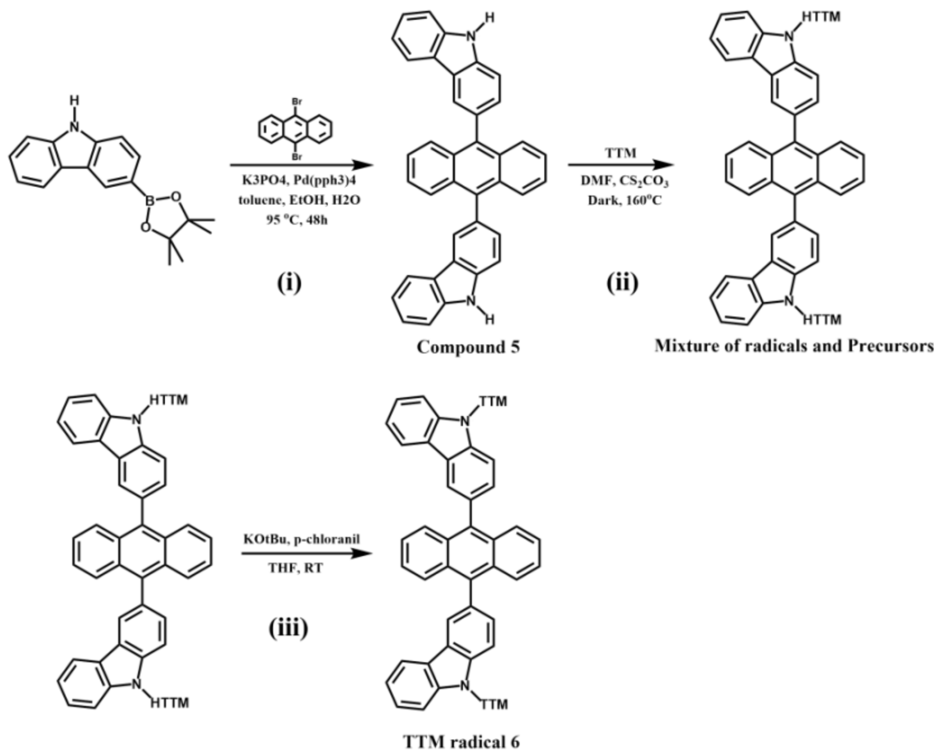

Scheme 3. Synthesis of (TTM-1Cz)$_2$-An (radical 6).

Scheme 3. (i): 3-(4,4,5,5-Tetramethyl-1,3,2-dioxaborolan-2-yl)carbazole (6.0 mmol) and 9,10-dibromoanthracene (2.0 mmol) were dissolved in a mixed solvent of toluene (24 mL), K_2CO3 aqueous solution (16 mL, 2 M) and ethanol (8 mL), then catalyst $Pd(PPh_3)_4$ (0.2 mmol) was added under argon atmosphere. The mixture was stirred at 95°C for 48 h under argon atmosphere and in the dark. Following this, the reaction mixture was cool to room temperature and filtered directly. The filter cake was washed three times with water and then recrystallized with tetrahydrofuran. Finally compound 5 was obtained as whitish solids.

Compound 5 white powder(0.55 g, 54%);1H NMR (500 MHz, DMSO) δ 11.62 – 11.56 (m, 2H), 8.23 (s, 2H), 8.17 (s, 2H), 7.77 (s, 2H), 7.69 (s, 4H), 7.60 (s, 2H), 7.51 – 7.47 (m, 2H), 7.46 – 7.42 (m, 2H), 7.39 (s, 4H), 7.19 – 7.15 (m, 2H).
(GC-MS) (m/z):508.74 (Fig. S5).

Scheme 3. (ii): TTM (3.0 mmol), compound 5 (1.0 mmol), anhydrous Cs_2CO_3 (3.0 mmol) were added in DMF (15 ml). The mixture was stirred at 160°c for 12 h under argon atmosphere and dark conditions. After the reaction mixture was cooled to room temperatures and poured into (0.2 M) hydrochloric acid solution; the precipitate was filtered and washed with water three times. The crude product was dissolved in dichloromethane and extracted with water and dichloromethane. The organic layer was dried over $MgSO_4$, and evaporated under vacuum. The crude product was purified by silica gel column chromatography (using petroleum ether: dichloromethane = 9:1, v/v). Mixtures of the desired radical 6 and its precursor compounds were obtained.

Radical 6 and its precursor: (0.1 g, 6.5%)

Scheme 3. (iii): Under argon atmosphere and dark conditions, the KOtBu (0.39 mmol.) was added to the THF solution (15ml) of radical 6 and its precursor mixtures (0.065 mmol.); The solution was stirred for 5 h at room temperature. Then tetrachloro-p-benzoquinone (0.52mmol.) was added and the solution was stirred for another 2 h. After the reaction finished, the solvent was removed under vacuum, and the crude product was purified by silica gel column chromatography (using petroleum ether: dichloromethane = 9:1, v/v). The desired radical 6 was obtained.

Radical 6 = (TTM-1Cz)$_2$-An: (0.08 g, 80%)
MS (GC-MS) (m/z):1543.50. (Fig. S6).
TOF-MS-ES$^+$: Calcd. for [$C_{76}H_{34}Cl_{16}N_2$]$^+$: 1536.7793. Found: *m/z* = 1536.7654. (Fig. S9).
EA: Calcd. for $C_{76}H_{34}Cl_{16}N_2$: C 59.19, H 2.22, N 1.82. Found: C 59.62, H 2.32, N 1.83.
ESR Spin density: 598 μM in nominal 300±60 μM solution (2.0±0.4 ≈ 2 paramagnetic centres per molecule).

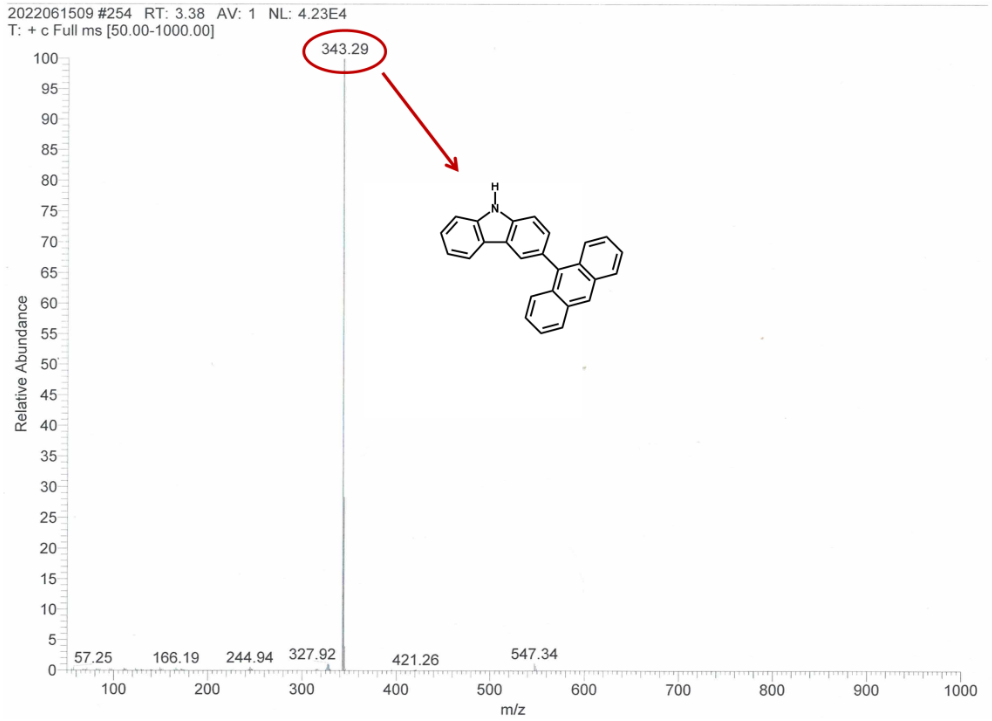

Figure S1. Mass Spectrum of Compound 1.

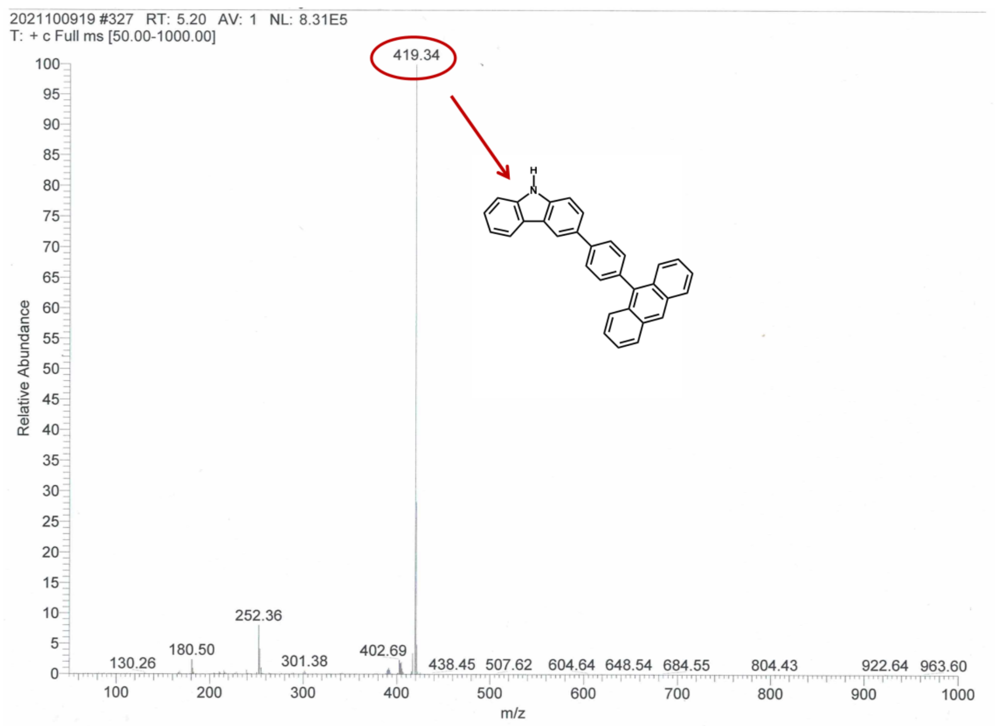

Figure S2. Mass Spectrum of Compound 2.

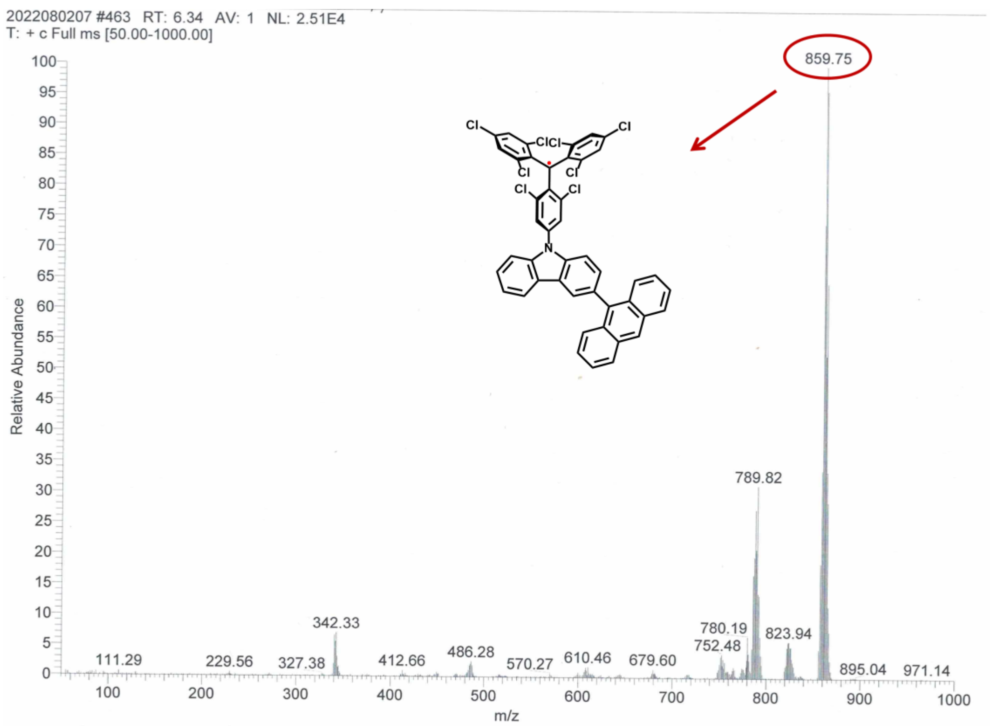

Figure S3. Mass Spectrum of TTM-1Cz-An (Radical 3).

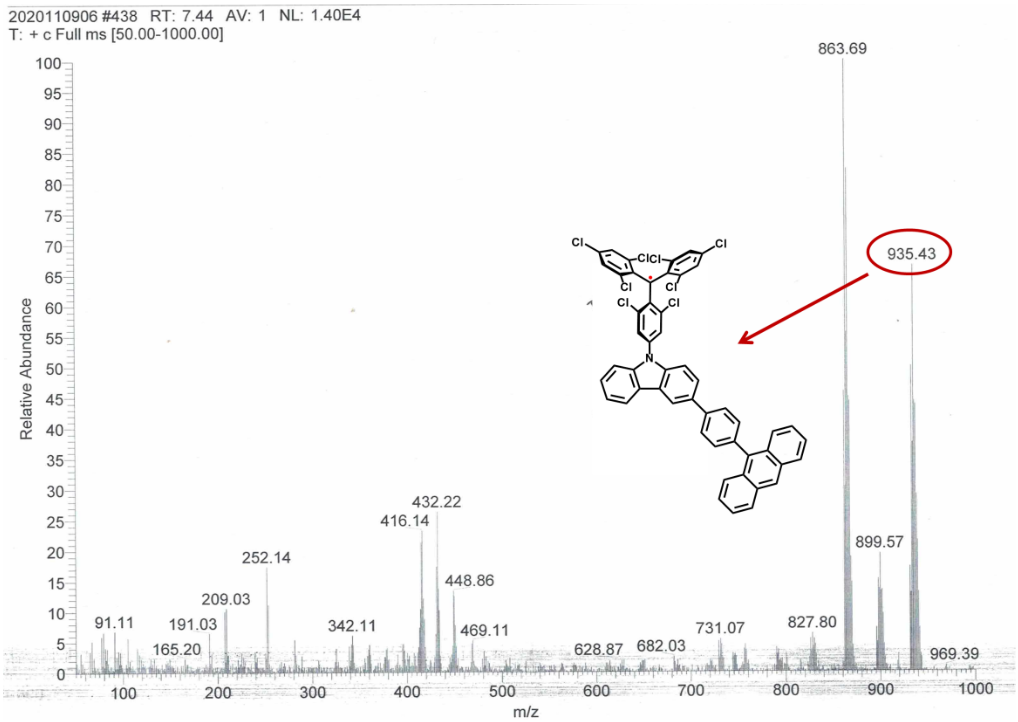

Figure S4. Mass Spectrum of TTM-1Cz-PhAn (Radical 4).

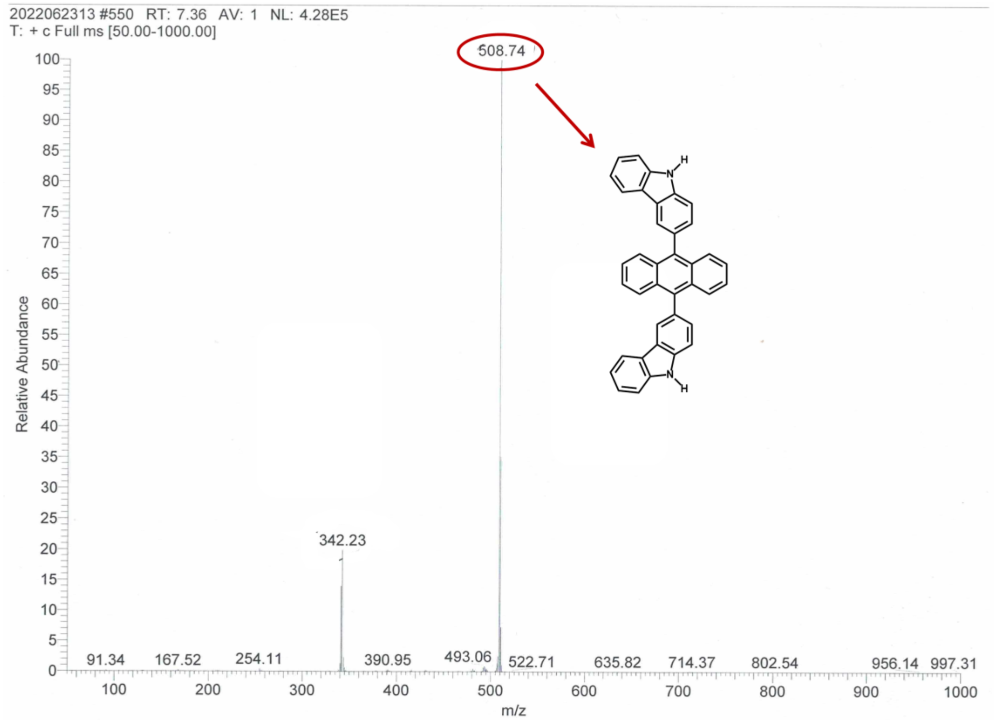

Figure S5. Mass Spectrum of Compound 5.

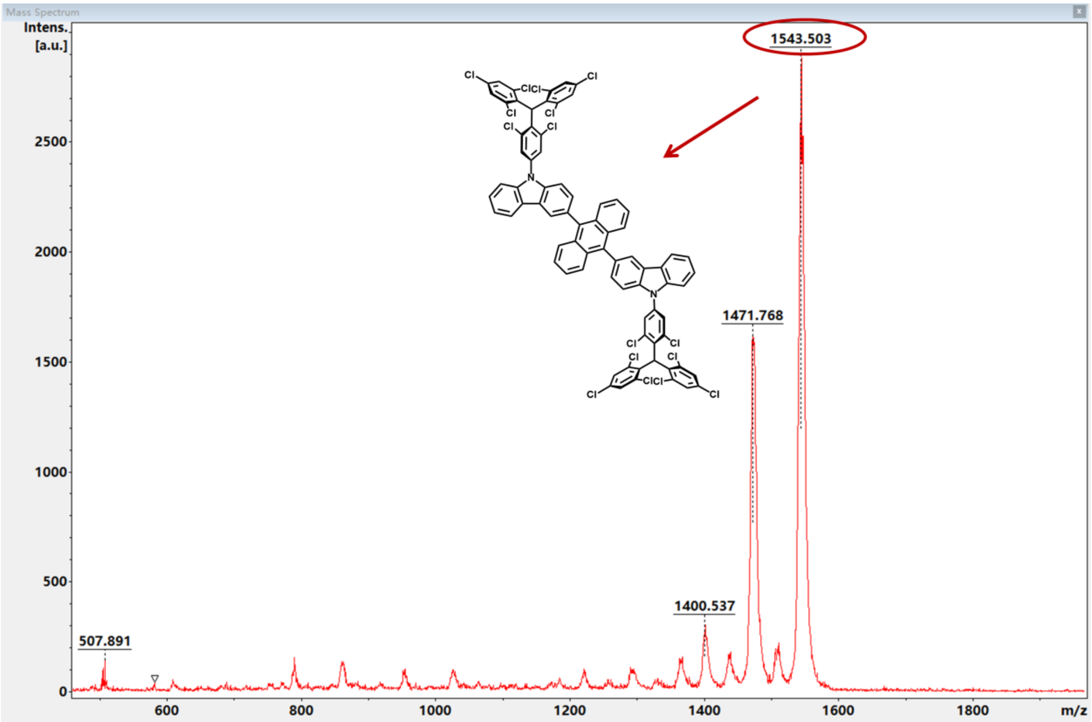

Figure S6. Mass Spectrum of (TTM-1Cz)$_2$-An (Compound 6).

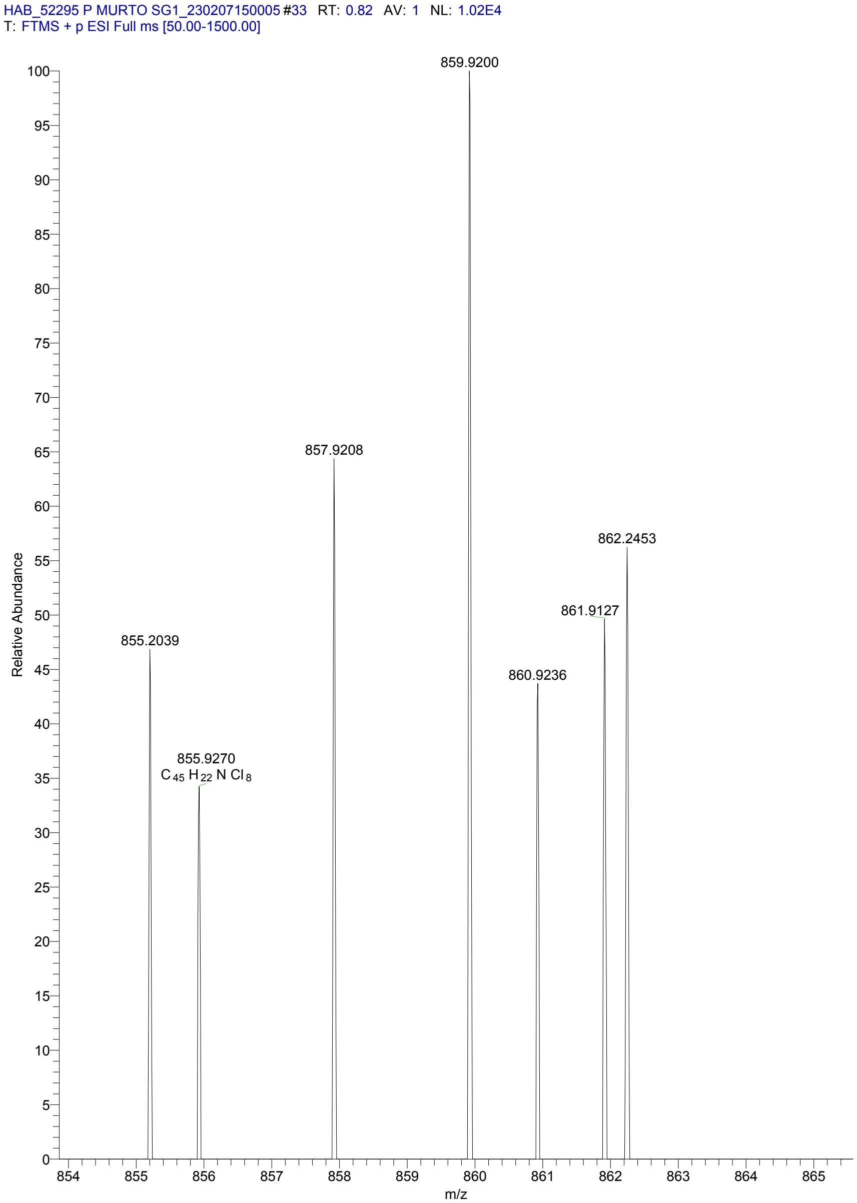

Figure S7. High Resolution Mass Spectrum of TTM-1Cz-An (Compound 3).

Tolerance = 50.0 PPM / DBE: min = -1.5, max = 50.0
Element prediction: Off
Number of isotope peaks used for i-FIT = 3

Monoisotopic Mass, Odd and Even Electron Ions
14 formula(e) evaluated with 1 results within limits (all results (up to 1000) for each mass)
Elements Used:
C: 0-51 H: 0-27 N: 0-1 Cl: 0-8

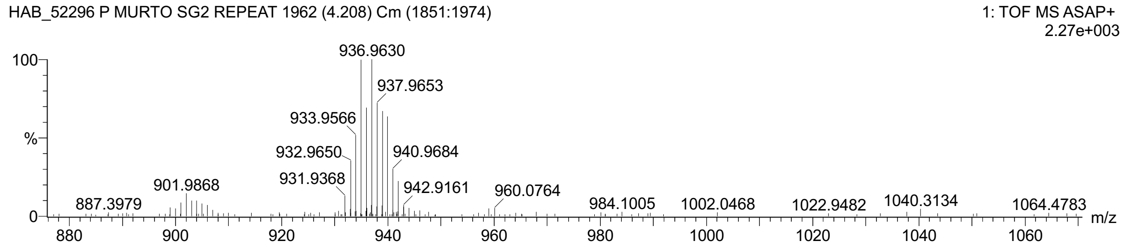

Minimum:				-1.5				
Maximum:		5.0	50.0	50.0				
Mass	Calc. Mass	mDa	PPM	DBE	i-FIT	Norm	Conf(%)	Formula
932.9650	932.9652	-0.2	-0.2	35.0	68.5	n/a	n/a	C51 H27 N Cl8

Figure S8. High Resolution Mass Spectrum of TTM-1Cz-PhAn (Compound 4).

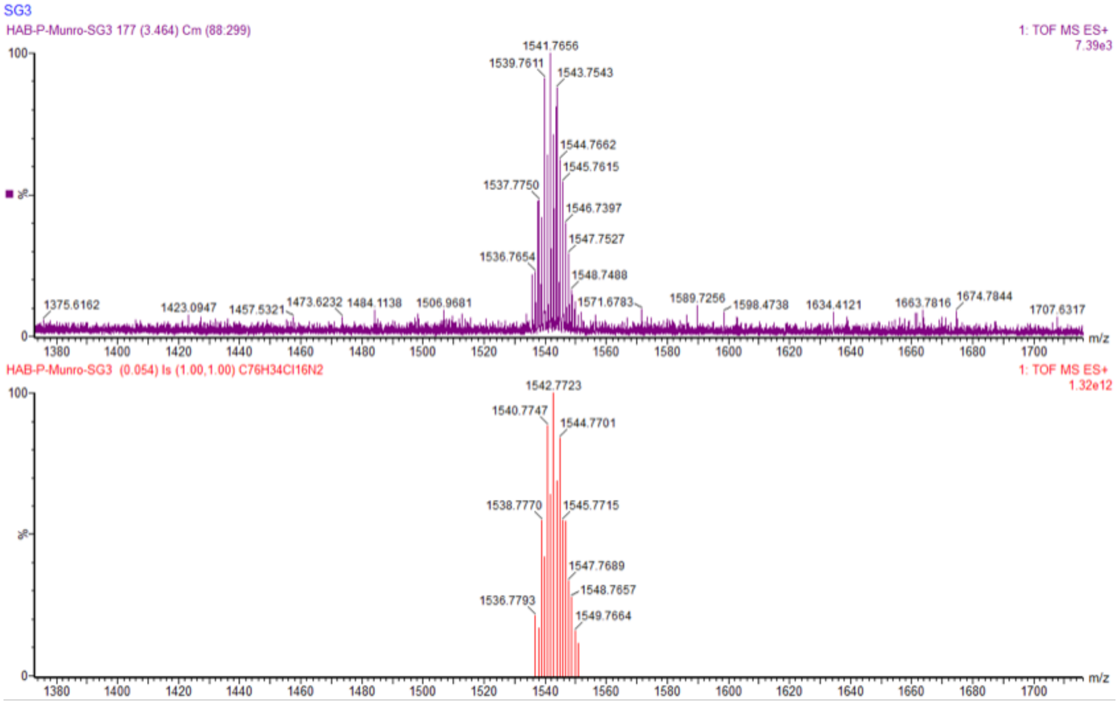

Figure S9. High Resolution Mass Spectrum of (TTM-1Cz)$_2$-An (Compound 6). Experimental data (top) and model (bottom) shown.

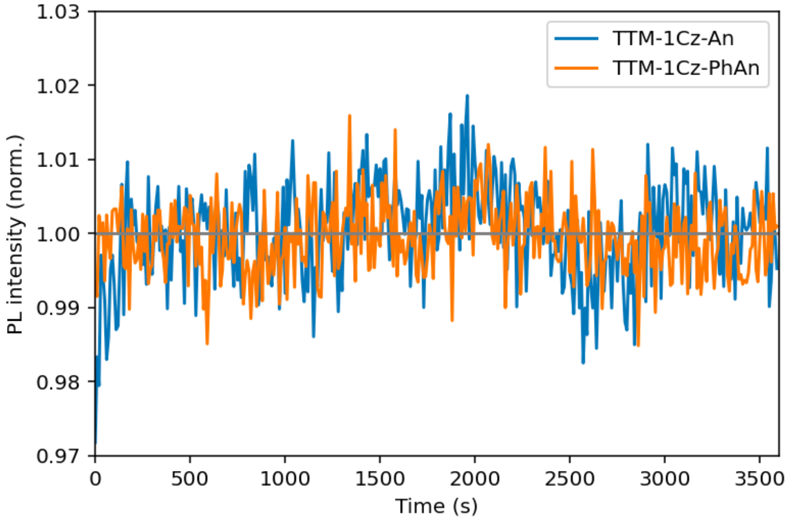

Figure S10. Photostability. Emission intensity integrated from 650-850 nm under cw 520 nm 6 mW excitation on encapsulated 5% radical in PMMA films at room temperature. Intensity corrected for the concurrently monitored pump laser power fluctuations. No PL decay is observed after 1 hour.

2. Supplementary experimental results

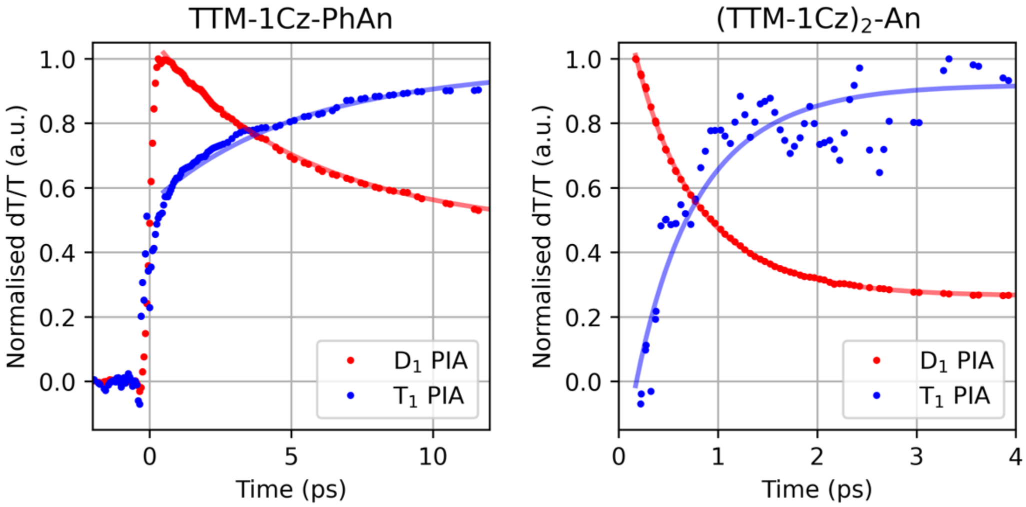

Figure S11. Ultrafast TA kinetics in 200 µM toluene solutions at 295 K photoexcited at 600 nm with fluence of 30 µJ/cm^2. Conditions as in Fig. 1d for TTM-1Cz-An. Kinetic slices extracted at 580 nm (D_1 PIA) and 430 nm (T_1 PIA). Solid lines are monoexponential fits described in the text.

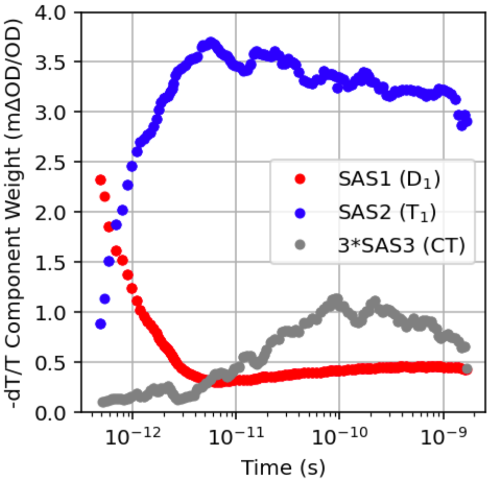

Figure S12. Population analysis in ps TA of 200 µM toluene solution of TTM-1Cz-An extracted from dataset presented in Extended Data Fig. 1. Spectral decomposition using a genetic algorithm,[47] extracts three Species Associated Spectra (SAS), which match the spectra expected for radical D_1 PIA, anthracene T_1 PIA and anthracene radical cation PIA signatures. The population of the component associated with the CT (scaled by a factor of 3 for clarity) peaks around 100 ps.

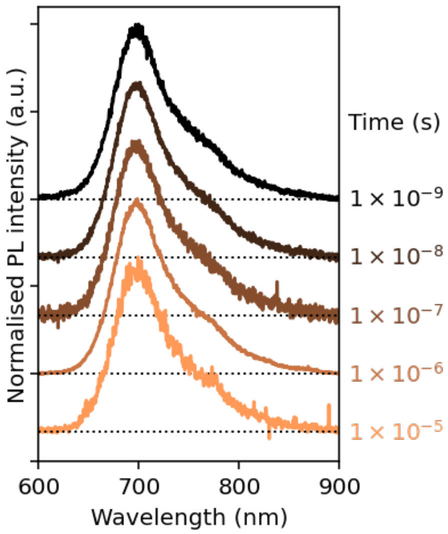

Figure S13. Time resolved emission spectra in 50 μM toluene solution of TTM-1Cz-An at 295 K following pulsed (~200 fs) 532 nm excitation with 4 μJ/cm^2 fluence. Emission lineshape is unchanged throughout the decay.

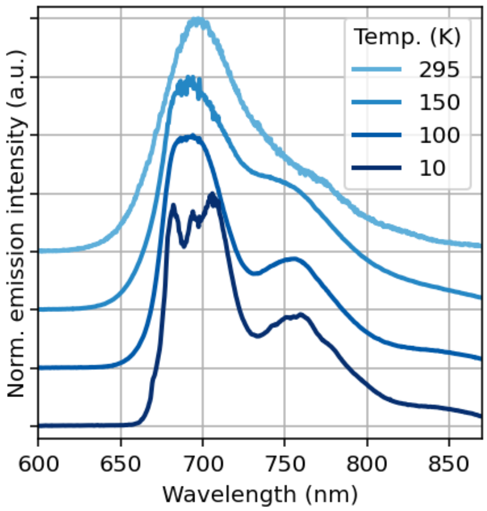

Figure S14. Temperature dependence of cw emission lineshape of TTM-1Cz-An. 200 μM toluene solutions under steady-state 532 nm excitation.

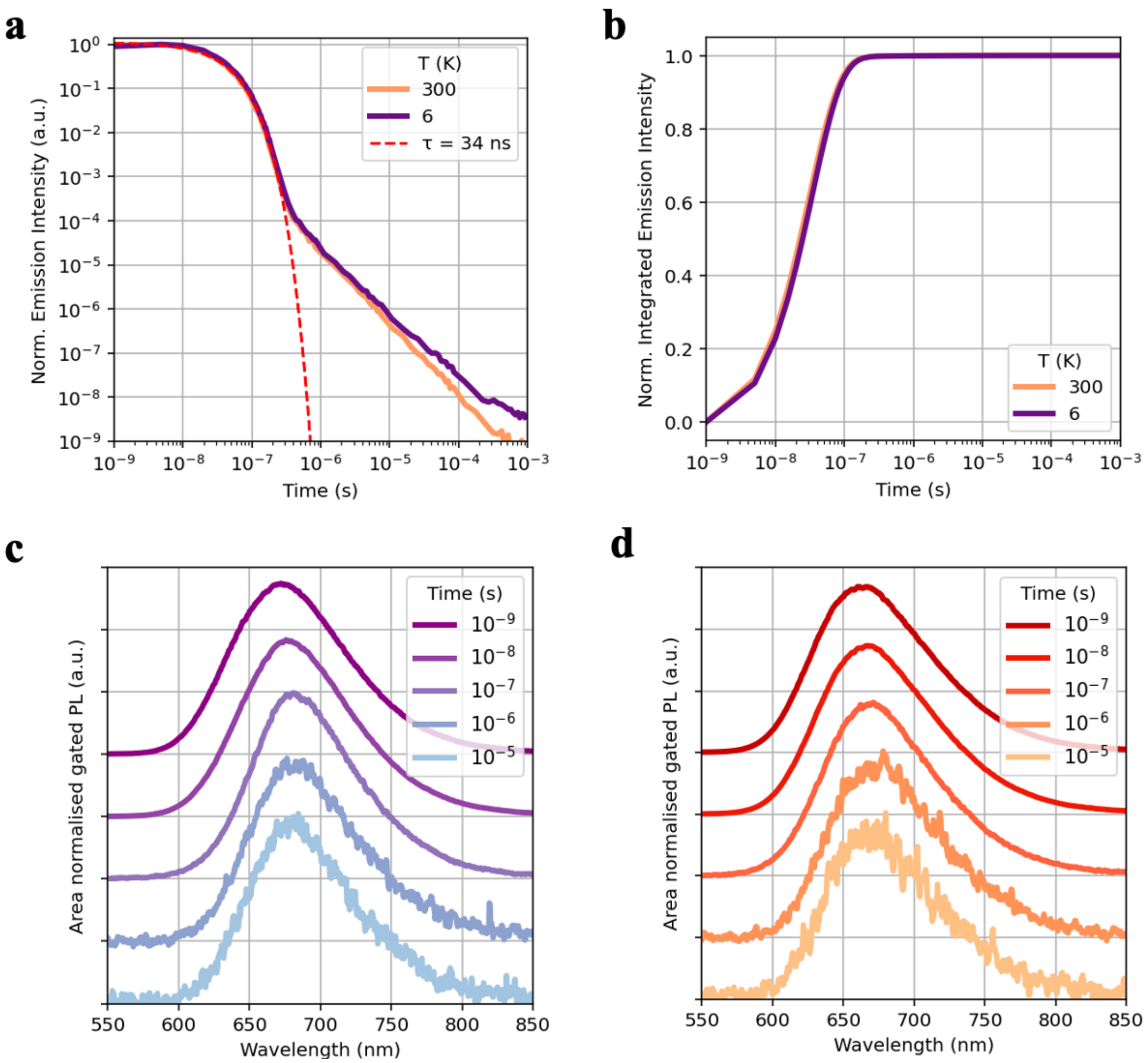

Figure S15. Time-resolved emission of 5% TTM-1Cz in PMMA films following pulsed (~200 fs) 520 nm excitation with fluence of 26 μJ cm^{-2}. **a,** Normalised emission kinetics in a 550-850 wavelength region. **b,** Normalised integrated emission kinetics extracted from panel a. Fitted monoexponential 34 ns lifetime captures 99.7% of the emission intensity. **c,** Normalised time gated emission spectra at 6 K. **d,** Normalised time gated emission spectra at 300 K. No temperature dependence observed in emission lineshape or kinetics.

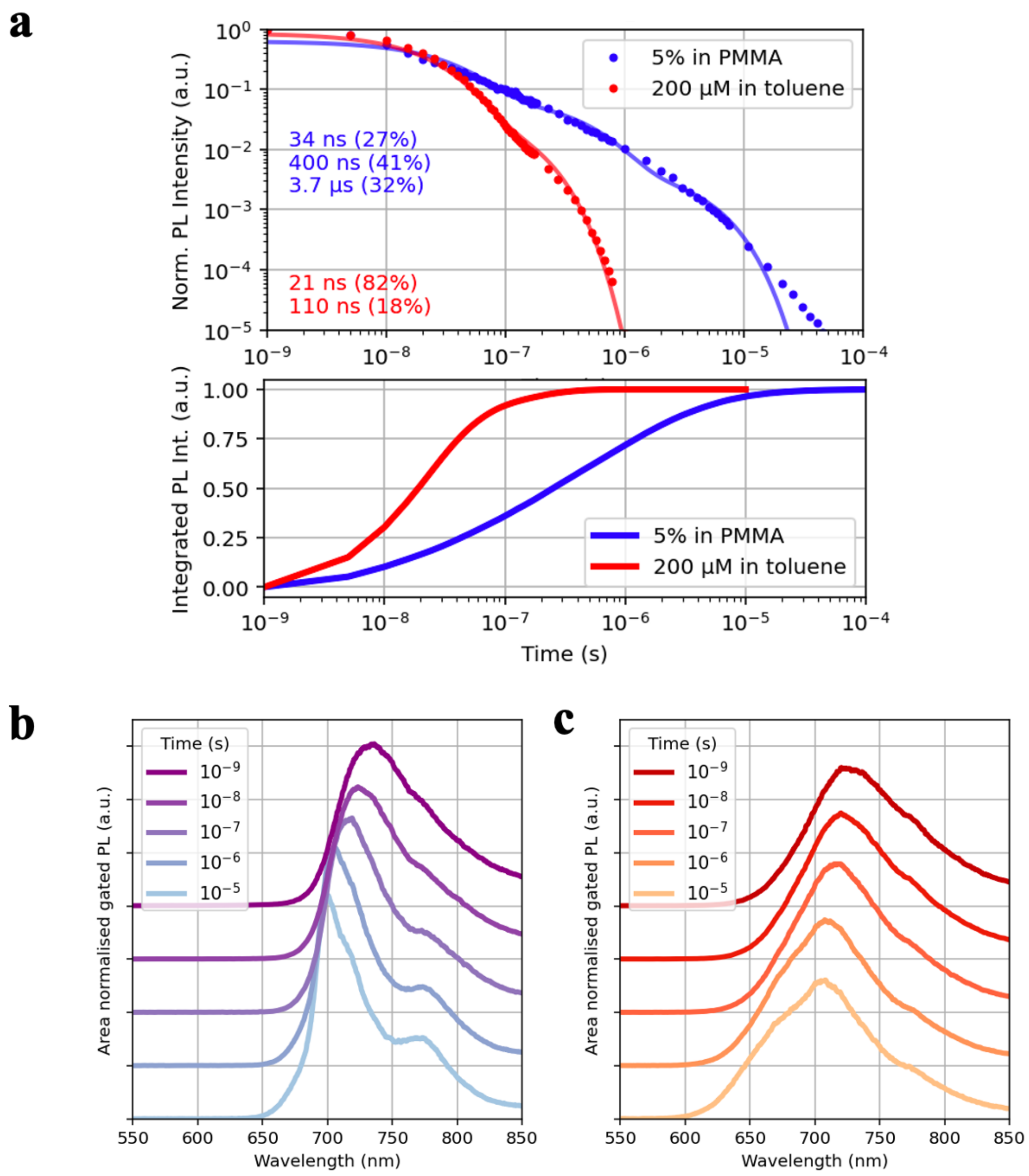

Figure S16. Time resolved emission of (TTM-1Cz)$_2$-An following pulsed (~200 fs) 530 nm excitation with fluence of 8.7 µJ cm^{-2}. **a,** Normalised emission kinetics summed within a 600-890 wavelength region (top) and integrated emission (bottom) for 200 µM toluene solution and 5% in PMMA film at 292 K. **b,** Normalised time gated emission spectra at 6 K showing structured phosphorescence at microsecond times. **c,** Normalised time gated emission spectra at 292 K.

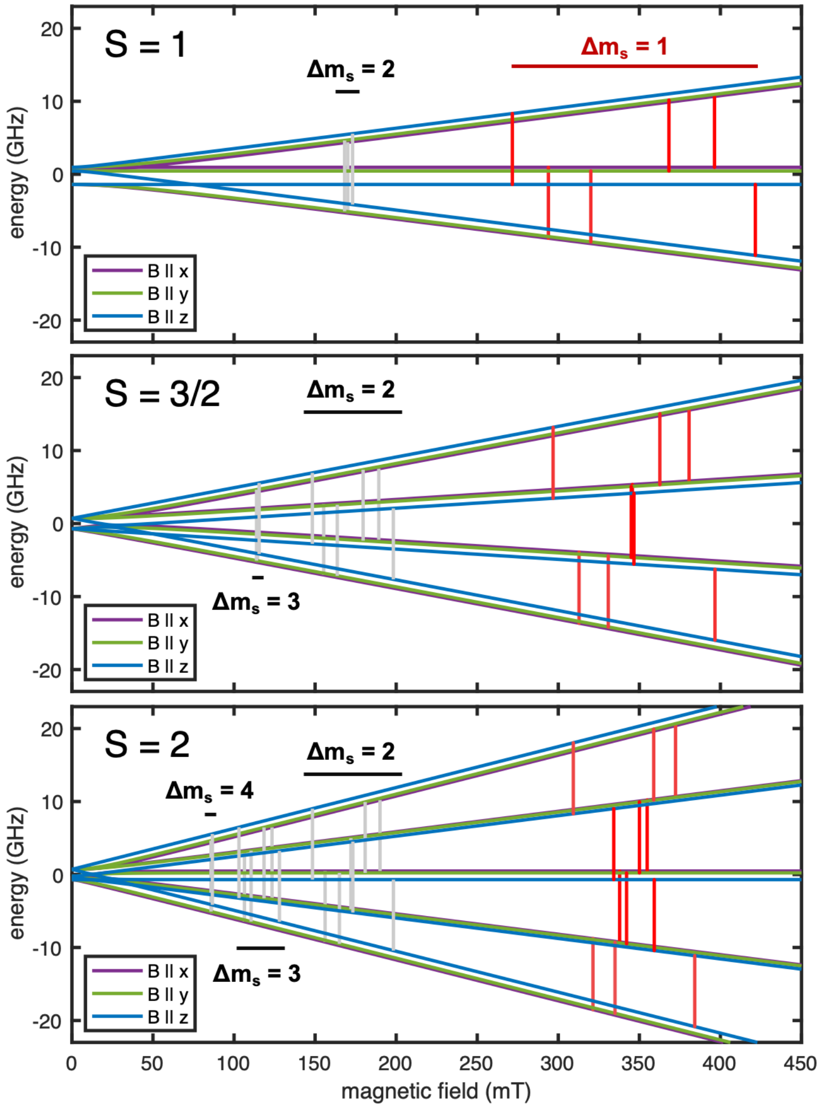

Figure S17: Calculated energy eigenstates along canonical orientations and resonant microwave transitions between sublevels at X-band (9.72 GHz). Zero field splitting and *g* values of anthracene triplets are used. The fully allowed FF transitions ($\Delta m_s = 1$) are shown in red. The width of the FF spectra narrows from ca. 150 mT for triplet (S = 1), to ca. 100 mT for quartet (S = 3/2), and ca. 75 mT for quintet (S = 2) signals. This matches well with the trESR spectra of R-A, and R-A-R.
The HF transitions (shown in grey) are orders of magnitude less intense than the FF transitions due to their forbidden nature. In the HF region of triplets, only a narrow $\Delta m_s = 2$ feature near $g = 4$ can be detected. For quartets, the $\Delta m_s = 2$ feature is significantly broader, spanning ca. 50 mT, and additionally a narrow $\Delta m_s = 3$ feature can be present near $g = 6$. The pattern of the quartet $\Delta m_s = 2$ feature resembles that of the triplet $\Delta m_s = 1$ feature. Similarly, the quintet $\Delta m_s = 2$ pattern resembles that of the quartet $\Delta m_s = 1$ feature, with a narrow central feature near $g = 4$ within a broader pattern with the same width as that of the quartet $\Delta m_s = 2$ transition. The observed HF transitions ($\Delta m_s = 2$ and 3 for R-A, and $\Delta m_s = 2$ for R-A-R) match predicted lineshapes, positions and widths.

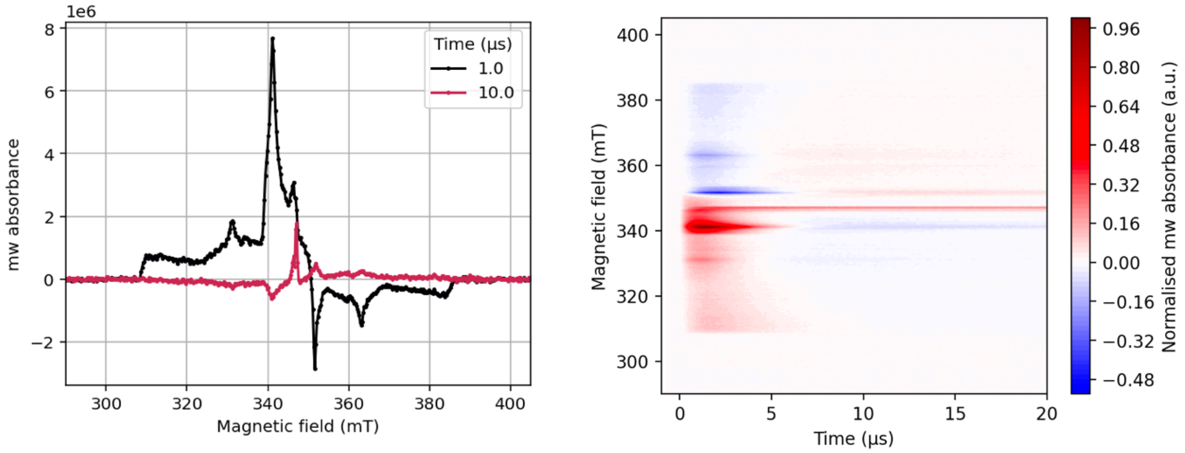

Figure S18. R-A-R cw trESR in the FF region at X-band. Frozen 200 µM toluene solution of $(TTM\text{-}1Cz)_2$-An at 80 K. Spectra acquired after optical excitation with 600 nm 500 µJ pulses.

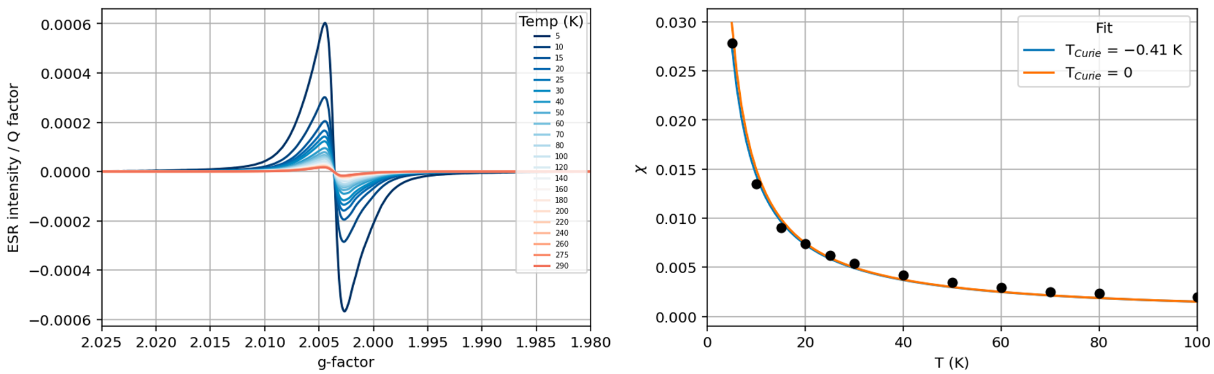

Figure S19. Temperature dependence of cw ESR intensity in R-A-R. 5% $(TTM\text{-}1Cz)_2$-An in PMMA film. Experiment performed at X-band without light excitation. Susceptibility (χ) extracted from double integral of the g = 2.00355 ESR lineshape after correcting for cavity quality (Q) factor which was measured after retuning at every temperature. Doublet-like behaviour is observed, as intensity increases upon lowering temperature.

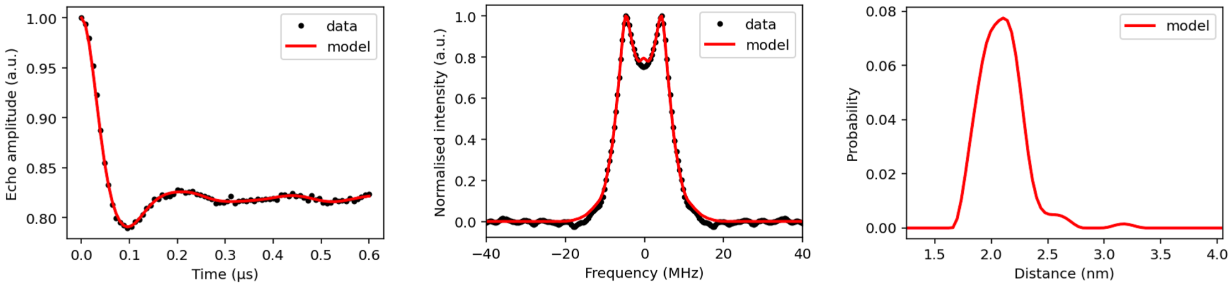

Figure S20. DEER on R-A-R ground state. Frozen 200 μM toluene solution of (TTM-1Cz)$_2$-An at 30 K. Experiment performed at field corresponding to g=2.00355 at Q-band (1215.8 mT) without light excitation. Analysis performed using DeerAnalysis.[48]

Additional experimental comments

a. Magnetophotoselection

Magnetophotoselection links the orientation of zero field splitting tensor axes relative to the optical transition dipole moment.[38] TTM-1Cz-An and TTM-1Cz-PhAn show an opposite magnetophotoselection effect on the intensity of the X and Y transitions. This matches the molecular conformations predicted by theoretical calculations, since the electric dipole generated with a 600 nm pulse lies along the TTM-carbazole axis for both molecules. In TTM-1Cz-An, the anthracene is twisted relative to the carbazole, while in TTM-1Cz-PhAn the anthracene and carbazole lie closer to the same plane, with the bridging phenyl linker orthogonal.

b. ESR modelling approach

We work in the partly coupled representation, taking $S_1 = 1$ (anthracene triplet) and $S_2 = 1/2$ (TTM ground state radical), as this allows us to accurately simulate the field region near $g = 2$. The g-factor and zero field splitting constants for the quartet state are transformed from their free radical and free triplet values according to: $g_Q = {}^{(g_R + 2g_T)}\!/_3$ and $D_Q = {}^{(D_T + D_{RT})}\!/_3$, where D_{RT} represents the magnetic dipolar-dipolar interaction between the radical and triplet within the quartet state. The radical-triplet dipolar coupling values extracted (D_{RT} ~70 MHz for TTM-1Cz-An) indicate weak radical-triplet dipolar coupling. In this strong exchange regime, a trip-quartet ($S = 3/2$) and trip-doublet ($S = 1/2$) spin level pair is formed and is well energetically separated at all magnetic fields accessed in our ESR experiments. The lineshape of transient ESR spectra in the strong exchange regime is not sensitive to the size of J.[6]

c. trODMR dynamics

Transient ODMR (trODMR) studies the system under continuous optical excitation, which is more representative of steady-state conditions at typical excitation densities and allows to probe the new equilibrium state to be probed upon microwave application.

We present a summary of the working principle of trODMR: Continuous optical excitation produces continuous PL. At a set magnetic field, microwave pulses are applied for a defined duration (MW on/off) resulting in a spin-dependent change of luminescence (ΔPL/PL). In contrast

to pulsed ODMR or EPR with the intention of coherent spin manipulation, the microwave pulse duration is longer to allow resonant effects to reach equilibrium intensities. After the microwave pulse, the system relaxes back to its previous equilibrium state of $\Delta PL/PL = 0$. Due to the direct recording mode of the optical signal, the data obtained have correct signs and amplitudes. This is in contrast to cw techniques that usually employ lock-in detection with microwave or magnetic field modulation, which results in time-averaged spectra with undetermined signal signs, and amplitudes depending on modulation frequency and lock-in phase.

Here trODMR measurements allow us to directly determine the sign of the ODMR spectra of R-A quartet states to be negative, corresponding to a reduction of PL under microwave resonant conditions.

The response of the PL to resonant conditions, *i.e.,* the ODMR signal rise time, is within the instrument response time, suggesting a fast coupling to phosphorescence. The signal decays exponentially to a steady state in resonant conditions with a time constant of 487 ± 22 μs. This slow establishment of the new steady state matches the long spin relaxation times observed in transient pulsed ESR.

d. Yield quantification

In our system we are able to estimate the high-spin state yield at room temperature using luminescence dynamics and PLQE, together with the triplet yield extracted from transient optical absorption. We have quantified the local triplet yield in TTM-1Cz-An by spectral deconvolution performed on the toluene TA dataset (Fig. S12). We find that 93% of the D_1 associated SAS signal is lost within the first 7 ps relative to the initial intensity.

As the triplet character generation occurs with an ultrafast lifetime of ca. 1 ps, it outcompetes any other excited state pathways. Noting that there is a single activation energy for all luminescence, the estimated quartet yield is: 0.93*0.32/0.41 = 73%, where the factor of 0.32/0.41 is the PLQE ratio of TTM-1Cz-An to TTM-1Cz that approximately accounts for non-radiative losses due to the additional excited states present in the pathway preceding emission in R-A compared to R.

3. Quantum chemical calculations

(i) Excited state calculations

State-averaged CASSCF calculations on the monoradical TTM-1Cz-An were performed for several active spaces (Table S1). In order to balance the computational cost and the accuracy of the results, an active space of 9 electrons in 7 orbitals, denoted as CAS(9,7), was chosen and NEVPT2 calculations were further carried out to include dynamical electron correlation (Table S2).

State	(5,5)	(7,6)	(9,7)	(11,8)	(13,9)	(15,10)
$^2[D_0T_1]$	2.73	2.72	2.85	2.88	2.86	2.83
$^4[D_0T_1]$	2.73	2.72	2.85	2.88	2.86	2.83
$^2[D_1S_0]$	3.74	3.63	3.67	3.87	3.38	3.43
^{2}CT	5.23	5.39	5.40	5.40	5.76	5.66
$^2[D_0S_1]$	5.25	5.47	5.50	5.30	5.47	5.61

Table S1: CASSCF / Def2-TZVP energies (in eV) as a function of the active space size.

State	Energy [eV]	Transition	Orbital composition	Oscillator strength
$^2[D_0T_1]$	2.26	$b \rightarrow j$	90%	1.5×10^{-7}
$^4[D_0T_1]$	2.26	$b \rightarrow j$	90%	/
$^2[D_1S_0]$	2.28	$c \rightarrow a$<br>$c \rightarrow k$	65%<br>15%	0.058
^{2}CT	2.34	$b \rightarrow a$	78%	0.010
$^2[D_0S_1]$	2.80	$b \rightarrow j$	79%	0.106

Table S2: Excited-state properties computed at the CAS(9,7) + NEVPT2 / Def2-TZVP level. Excited states above 3 eV are not included in the table. The transition labelling refers to Figure S21.

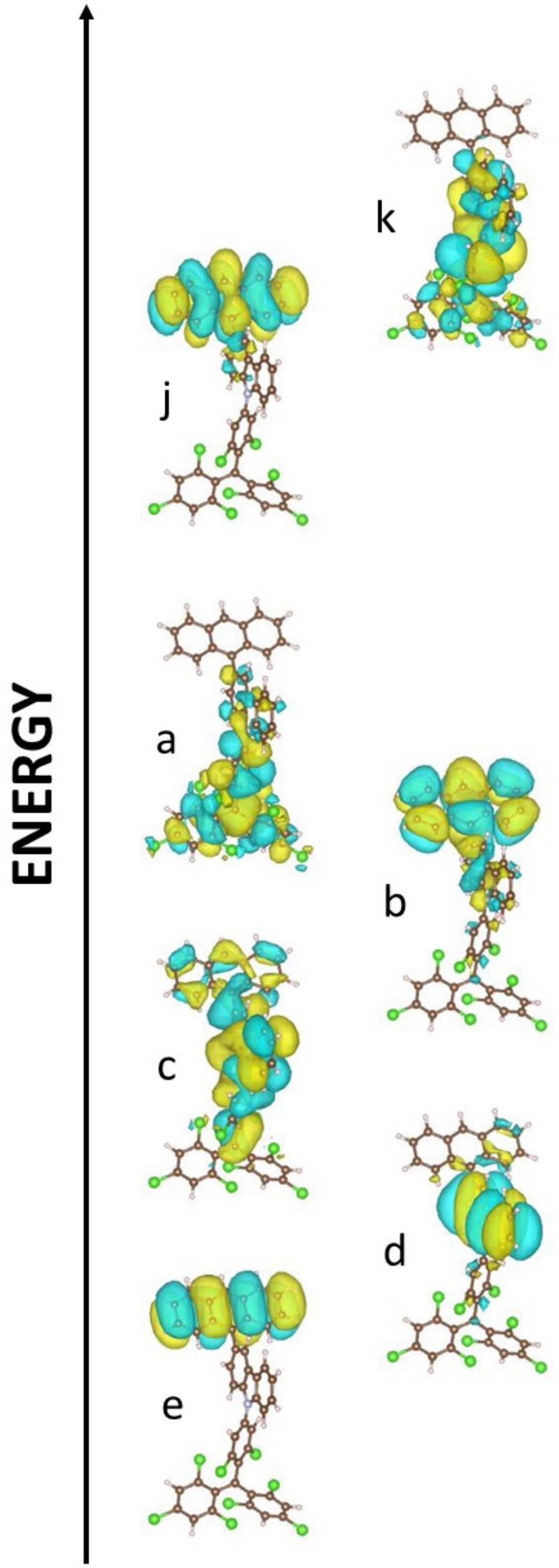

Figure S21: TTM-1Cz-An molecular orbitals obtained at the CAS(9,7) / Def2-TZVP level.

Referring to Figure S21, at the ground-state $^2[D_0S_0]$, the unpaired electron resides on the singly occupied molecular orbital (MO) *a* localised on the TTM moiety, while the MOs *b*, *c, d* and *e* are doubly occupied, and *j* and *k* are empty. The excited states $^{2/4}[D_0T_1]$ are predominantly described by a single excitation between MOs localised on the anthracene (An) (from *b* to *j*). These excited states involve the coupling between the chromophore pure triplet and the unpaired electron on the TTM moiety either in its spin-up ($m_s = +1/2$) or spin-down ($m_s = -1/2$) configuration, leading to an overall quartet or doublet state, respectively. It is interesting to notice that orbital *b* shows some degree of delocalization over the carbazole (Cz) group. The $^2[D_1S_0]$ state is described by two single excitations from the Cz group to the TTM moiety (from *c* to *a* and *k*), with some spread of the electronic wave function over the An core. According to NEVPT2 energies, this state is above the $^{2/4}[D_0T_1]$ states by 13 meV. Interestingly, an intramolecular doublet charge-transfer (^{2}CT) state is present 72 meV above the $^{2/4}[D_0T_1]$ states. This ^{2}CT state displays a transition from the An to the TTM moiety (from *b* to *a*, weight of 78%), with some minor contribution on the Cz unit. At last, the singlet counterpart of the pure triplet on the An core, $^2[D_0S_1]$, lies 0.54 eV above the $^{2/4}[D_0T_1]$ states, as expected from the large exchange interaction between singlet and triplet excited states in π-conjugated linear acenes.[49]

The conformation of the monoradical TTM-1Cz-An molecule is dictated by two dihedral angles: that between the TTM and the Cz and that between the Cz and the An. In Figure S22, the TTM-Cz torsion angle profile (top, green dashed frame) shows a minimum at 52°, while the Cz-An one (bottom, blue dashed frame) at 78°. The fully DFT-optimised ground-state $^2[D_0S_0]$ structure displays the same values for the two torsion angles (52° and 78°, respectively). Considering a threshold of k_BT, at room temperature (RT) corresponding to ~0.6 kcal/mol (red dashed line), TTM-1Cz-An appears to be quite flexible with the TTM-Cz dihedral angle varying between 40° and 65°, while the Cz-An dihedral angle could vary between 60° and 90° and 90° and 120° (since the energy profile is symmetric, besides being flat). It turns out that at RT the molecule can experience a broad distribution of possible conformers and, therefore, a broad energetic landscape of the excited states (Tables S3-S5).

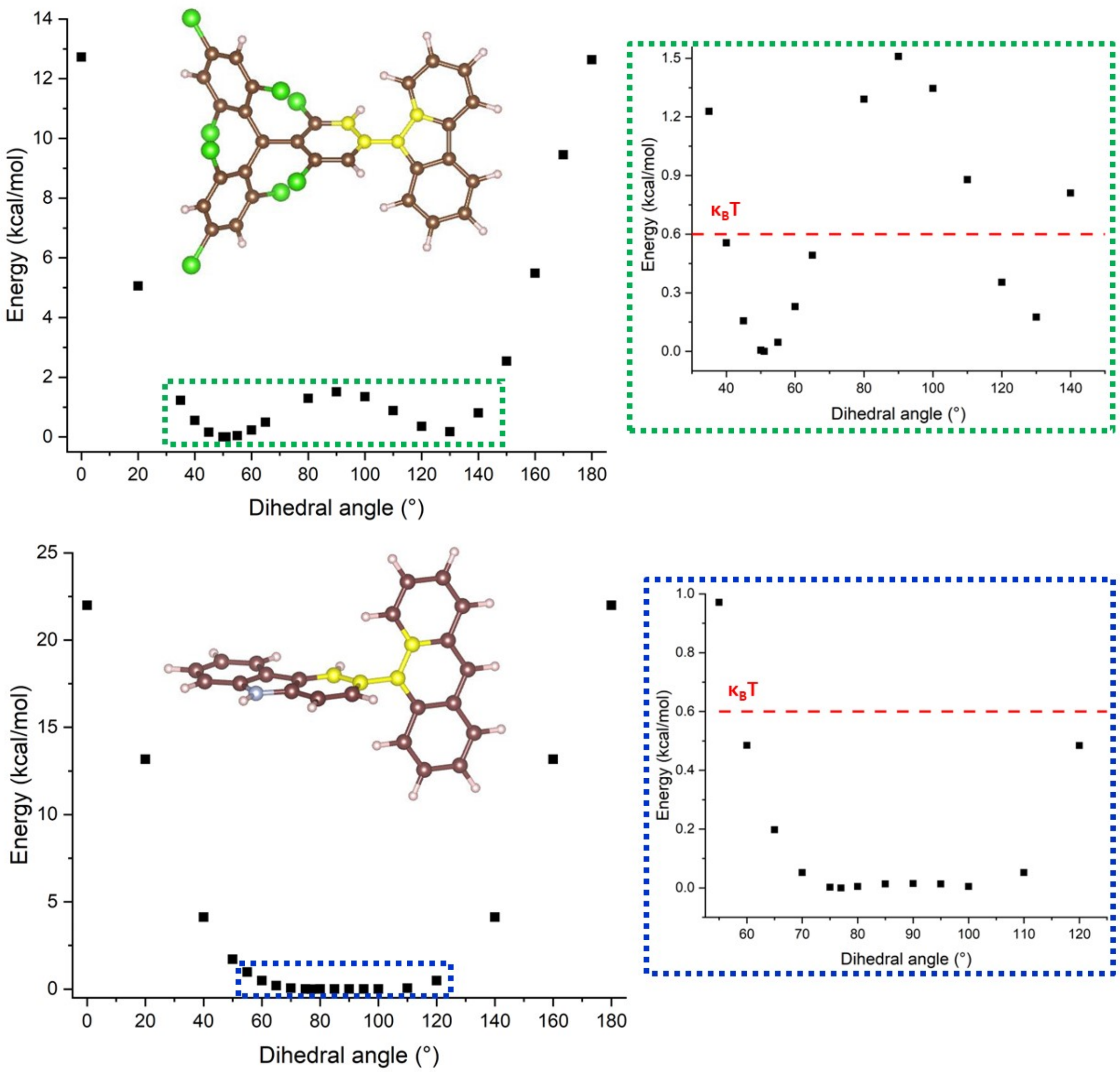

Figure S22: Torsion angle energy profile computed at the (UKS) DFT ωB97X-D/6-31G(d,p) level for the TTM-Cz (top) and Cz-An (bottom) dihedral angle, with insets of the region where the minimum lies.

From this analysis eight other conformers, different from the optimised ground-state structure (52-78), were built: (40-60), (40-78), (40-90), (52-60), (52-90), (65-60), (65-78), (65-90), where the numbers refer to the TTM-Cz and the Cz-An dihedral angles, respectively. On each generated structure, a CAS(9,7) + NEVPT2 / Def2-TZVP calculation was carried out. Several properties, reported in the underlying tables, were analysed upon changing the torsion angles of the molecule. The oscillator strength of the $^2[D_0T_1]$ increases by 3-4 orders of magnitude when reducing the Cz-An torsion angle from 90° to 60°, while decreasing the TTM-Cz from 65° to 40° only slightly affects it (Table S3). Besides, varying both dihedral angles do not dramatically influence the oscillator strength of the $^2[D_1S_0]$ state (Table S4). The spin-orbit coupling (SOC) between $^2[D_0T_1]$

and $^4[D_0T_1]$ is hardly affected by a change in the two dihedral angles in line with the very similar nature for both states (Table S6). While the SOC between $^2[D_1S_0]$ and $^4[D_0T_1]$ increases up to 0.044 cm^{-1} for the (65-60) conformation (Table S7), it remains much smaller than that between ^{2}CT and $^4[D_0T_1]$ due to the very different nature of these two states (Table S8).

		**Cz-An dihedral angle**		
		**60°**	**78°**	**90°**
**TTM-Cz dihedral angle**	**40°**	2.24 (1.4×10^{-5})	2.28 (2.2×10^{-7})	2.26 (9×10^{-9})
	**52°**	2.24 (1.6×10^{-5})	2.26 (1.5×10^{-7})	2.26 (1×10^{-8})
	**65°**	2.23 (6.8×10^{-6})	2.25 (7.5×10^{-8})	2.26 (7×10^{-9})

Table S3: Energy and oscillator strength (in parenthesis) of the $^2[D_0T_1]$ state as a function of the two dihedral angles.

		**Cz-An dihedral angle**		
		**60°**	**78°**	**90°**
**TTM-Cz dihedral angle**	**40°**	2.16 (0.042)	2.20 (0.038)	2.16 (0.041)
	**52°**	2.23 (0.035)	2.28 (0.058)	2.28 (0.058)
	**65°**	2.31 (0.048)	2.37 (0.049)	2.38 (0.049)

Table S4: Energy and oscillator strength (in parenthesis) of the $^2[D_1S_0]$ state as a function of the two dihedral angles.

		**Cz-An dihedral angle**		
		**60°**	**78°**	**90°**
**TTM-Cz dihedral angle**	**40°**	2.53 (0.021)	2.43 (0.007)	2.14 (8.4×10^{-5})
	**52°**	2.72 (0.022)	2.34 (0.010)	2.29 (1.3×10^{-5})
	**65°**	2.66 (0.047)	2.35 (0.004)	2.30 (4.0×10^{-5})

Table S5: Energy and oscillator strength (in parenthesis) of the ^{2}CT state as a function of the two dihedral angles.

		**Cz-An dihedral angle**		
		**60°**	**78°**	**90°**
**TTM-Cz dihedral angle**	**40°**	0.002	0.001	0.000
	**52°**	0.002	0.001	0.000
	**65°**	0.002	0.001	0.000

Table S6: Spin-orbit coupling (in cm^{-1}) between the $^2[D_0T_1]$ and $^4[D_0T_1]$ states as a function of the two dihedral angles.

		Cz-An dihedral angle		
		**60°**	**78°**	**90°**
**TTM-Cz dihedral angle**	**40°**	0.026	0.012	0.001
	**52°**	0.022	0.013	0.001
	**65°**	0.044	0.016	0.001

Table S7: Spin-orbit coupling (in cm^{-1}) between the $^{2}[D_1S_0]$ and $^{4}[D_0T_1]$ states as a function of the two dihedral angles.

		Cz-An dihedral angle		
		**60°**	**78°**	**90°**
**TTM-Cz dihedral angle**	**40°**	0.181	0.158	0.139
	**52°**	0.165	0.140	0.127
	**65°**	0.165	0.130	0.102

Table S8: Spin-orbit coupling (in cm^{-1}) between the ^{2}CT and $^{4}[D_0T_1]$ states as a function of the two dihedral angles.

For the R-A-R biradical $(TTM\text{-}1Cz)_2$-An, we performed CASSCF + NEVPT2 calculations on the DFT-optimised ground-state $^{3}[D_0S_0D_0]$ structure, by using an active space of 8 electrons in 8 orbitals and a smaller basis set as Def2-SVP in order to reduce the computational demands. In these calculations, only 1 quintet, 5 triplet and 4 singlet states were included in the state-averaged CASSCF procedure. The obtained MOs are shown in Figure S23, where the orbitals are labelled in the same spirit as in Figure S22, but with the difference that in Figure S23 orbital *d* and *e* are missing because not included in the CAS(8,8). The four $[D_0T_1D_0]$ excited states can be described by an electronic transition from MOs localized on the An core (from *b* to *j*), similar as for the R-A monoradical case. In fact, they involve the coupling between the An triplet and the two electrons, each residing on the radical TTM moiety (orbital *a'* and *a''*). The two electrons can be found once both in a spin-up (m_s = +1/2) configuration yielding the overall $^{5}[D_0T_1D_0]$ quintet state, twice in a spin-up and spin-down configuration yielding two $^{3}[D_0T_1D_0]$ triplet states and once both in a spin-down (m_s = -1/2) configuration yielding a $^{1}[D_0T_1D_0]$ singlet state (cf. Figure 4d-e in the main text).

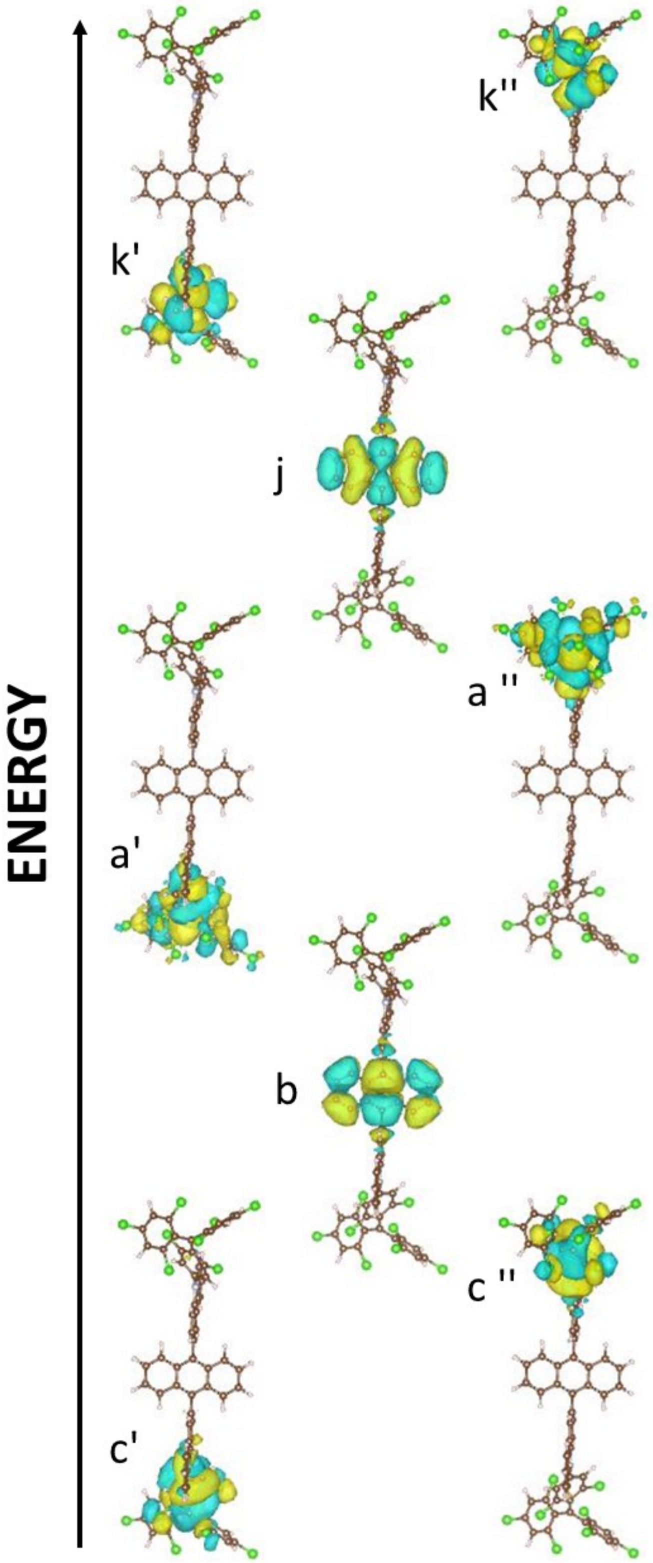

Figure S23: $(TTM\text{-}1Cz)_2$-An molecular orbitals obtained at the CAS(8,8) / Def2-SVP level.

(ii) Magnetic properties

Magnetic properties of the monoradical TTM-1Cz-An and TTM-1Cz-PhAn, such as the zero-field splitting (ZFS) and the g-tensor (Table S9), were calculated on the UKS optimised $^4[D_0T_1]$ structure, using B3LYP and the Def2-TZVP basis set, including the scalar relativistic zero-th order regular approximation (ZORA) Hamiltonian, as implemented in the ORCA 4.2 code. In particular, for the calculation of the D tensor, the first-order term, that is the spin-spin (dipolar) interaction, was computed by using the unrestricted natural orbitals (UNOs), while for the second-order term, that is the spin-orbit interaction, the mapping of the SOC contribution to the D tensor was performed following van Wüllen's method for the pre-coefficients of the angular momenta integrals, as shown in a previous work.[50] The calculation of the g-tensor was done exploiting the Gauge Including Atomic Orbitals (GIAOs) approach.
In the computation of the ZFS tensor, the following convention was chosen:

$$D = D_{zz} - \frac{1}{2}(D_{xx} + D_{yy})$$

$$E = \frac{1}{2}(D_{xx} - D_{yy})$$

so that $-\frac{1}{3} \leq \frac{E}{D} \leq +\frac{1}{3}$. For both monoradicals, the D parameter of the ZFS tensor shows the same sign ($D > 0$) and is in quantitative agreement with the experimental value, while the E parameter sign cannot be unambiguously chosen. The isotropic g value is equivalent for the two systems.

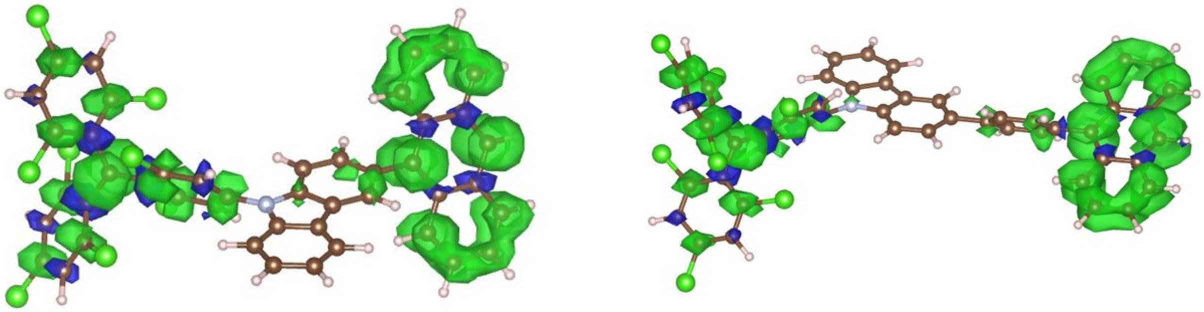

Figure S24: Spin density distribution for the quartet $^4[D_0T_1]$ state in TTM-1Cz-An (left) and TTM-1Cz-PhAn (right).

	**TTM-1Cz-An**	**TTM-1Cz-PhAn**
D (MHz)	753	730
$\|\|E\|\|$ (MHz)	118	160
g_x	2.002300	2.002273
g_y	2.002808	2.002668
g_z	2.003666	2.003800
g_{iso}	2.002925	2.002914

Table S9: ZFS parameters and g-tensor for the two radicals in the quartet state.

(iii) Electronic couplings calculations

At RAS-CI(11,10) level, the excited states are defined by single-electron transitions between orbitals within the α (m_s = +1/2) and the β (m_s = -1/2) electron subspace. The overall nature of a given excited state is determined considering the combination of these transitions (Table S10). The first excited state is characterised by three electronic configurations: the first one leaves the α-subspace unaltered, while in the β-subspace a transition from orbital *b* to *j* localised on the An core is occurring. The second configuration is characterised by the transition from orbital *a* to *j* in the α-subspace (from the TTM to An), and by the transition from orbital *b* to *j* in the β-subspace (from An to TTM); overall, the combination of these two results in a transition localised on the An. The third configuration is characterised by a transition from orbital *b* to *j* in the α-subspace, which is localised on the An, while the β-subspace remains unaltered. It follows that all the three configurations characterizing this state are localised on the An core and can be associated to the $^2[D_0T_1]$ state. The same analysis can be easily done to define the nature of the other excited states. The $^2[D_0S_1]$ state was identified considering the sign of the expansion coefficients of the two electronic configurations. As shown in Table S10, the two electronic configurations are the same as the first and the third one characterizing the $^2[D_0T_1]$ state. In this latter case, the sign of the coefficients defines a "+" linear combination (*i.e.,* a triplet CSF), while for $^2[D_0S_1]$ the sign of the coefficients defines a "-" linear combination (*i.e.,* a singlet CSF).
It is worth highlighting that the RAS-CI formalism introduces the dynamical correlation only partially. As such, the relative energy between the excited states and their energy order does not recover the ones obtained at NEVPT2 level (*e.g.,* the $^2[D_0S_1]$ in RAS-CI appears lower in energy than $^2[D_1S_0]$ and ^{2}CT, contrary to NEVPT2).

State	Energy [eV]	α spin-orbital composition	β spin-orbital composition	Coefficient
$^2[D_0T_1]$	3.20	/<br>*a* → *j*<br>*b* → *j*	*b* → *j*<br>*b* → *a*<br>/	0.40<br>0.81<br>0.40
$^2[D_0S_1]$	4.80	/<br>*b* → *j*	*b* → *j*<br>/	0.66<br>-0.66
$^2[D_1S_0]$	5.08	/<br>*a* → *k*<br>*a* → *particle*	*c* → *a*<br>*c* → *a*<br>*c* → *a*	0.65<br>0.34<br>0.33
^{2}CT	5.68	/<br>/	*b* → *a*<br>*d* → *a*	0.70<br>0.33

Table S10: Doublet excited states energies (in eV), α and β spin-orbitals composition and expansion coefficients obtained at RAS-CI(11,10) / Def2-SVP level.

The diabatization procedure was applied to doublet excited states in order to compute the electronic couplings between $^2[D_1S_0]$, $^2[D_0T_1]$ and ^2CT diabatic states. For the identification of the diabatic state nature we relied on the analysis of the attachment and detachment density distribution on the TTM, Cz and An fragment (Figure S25).

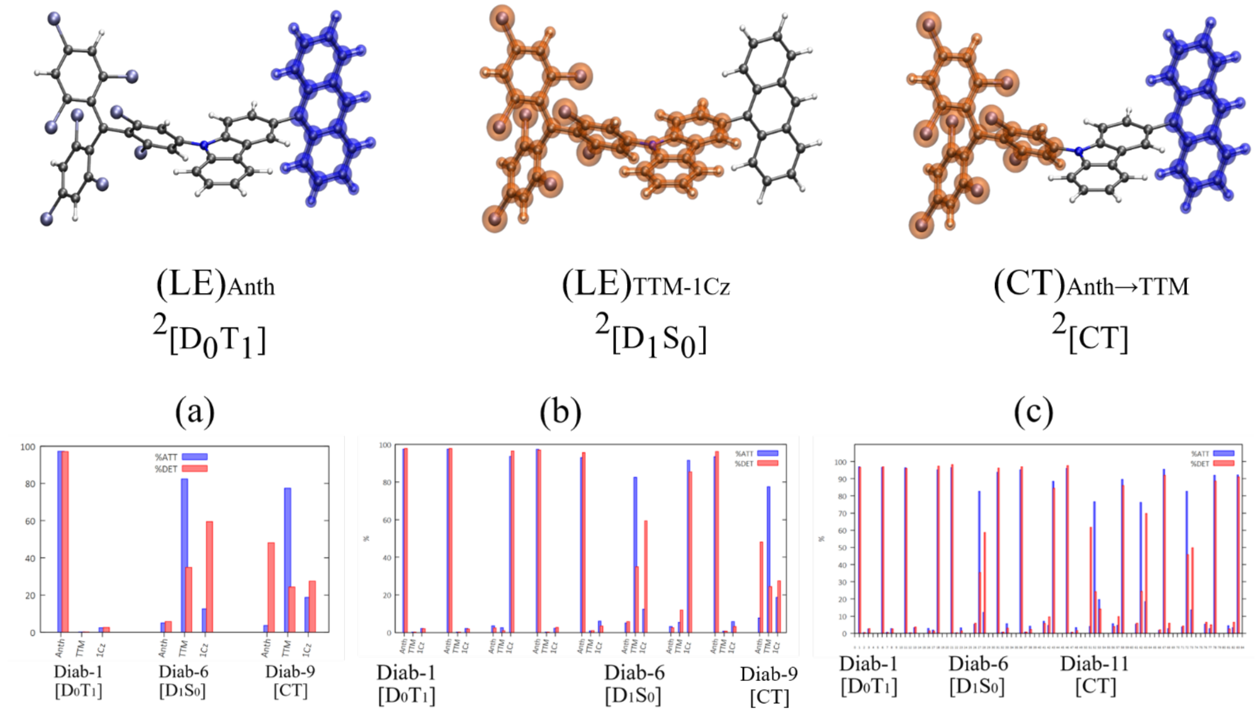

Figure S25: Target diabatic states for TTM-1Cz-An and percentage of attachment (red) and detachment (blue) densities localised on the An, TTM and Cz fragment for the $^2[D_1S_0]$, $^2[D_0T_1]$ and ^2CT diabatic states within the 3x3 (a), 9x9 (b) and 17x17 (c) diabatization.

By increasing the diabatization size, the magnitude of the diabatic state energies and the electronic couplings slightly changes (Table S11 and S12), with the coupling between the localised state ($^2[D_1S_0]$ and $^2[D_0T_1]$) and the ^2CT being the highest. Therefore, and hereafter we will rely on the 9x9 diabatization scheme, showing a good trade-off between computational cost and accuracy.
We also performed the 9x9 diabatization on two other conformers, where the Cz-An dihedral angle was set to 60° (52-60) and 90° (52-90) (Figure S27 and S28, respectively). At 60° the nature of the $^2[D_0T_1]$ and $^2[D_1S_0]$ undergoes a negligible modulation, with the percentage of the attachment and detachment densities on the three fragments remaining similar for all the conformers. On the contrary, by increasing the torsion angle to 90°, there is an increase of the detachment density on the An core along with a decrease of the detachment distribution on the Cz for the ^2CT state, resulting in a more prominent CT-character. Interestingly, at 90° the coupling between $^2[D_1S_0]$ and $^2[D_0T_1]$ (*i.e.,* a direct coupling) slightly changes, while a more drastic decrease is observed for the couplings involving the ^2CT diabatic state (Table S13).

State	3x3	9x9	17x17
$^2[D_0T_1]$	3.20	4.05	4.51
$^2[D_1S_0]$	5.08	5.09	5.16
^2CT	5.68	5.67	5.71

Table S11: $^2[D_1S_0]$, $^2[D_0T_1]$ and ^2CT diabatic states energies (in eV) as a function of the diabatization size computed at RAS-CI(10,11) / Def2-SVP level for the DFT-optimised ground-state (52-78) structure.

Coupled states	3x3	9x9	17x17
$\langle [D_0T_1] \vert \hat{H} \vert [D_1S_0] \rangle$	-4	-3	-4
$\langle [D_0T_1] \vert \hat{H} \vert [CT] \rangle$	-16	-10	-2
$\langle [D_1S_0] \vert \hat{H} \vert [CT] \rangle$	92	96	79

Table S12: Electronic couplings (in meV) between the $^2[D_1S_0]$, $^2[D_0T_1]$ and ^2CT diabatic states as a function of the diabatization size computed at RAS-CI(10,11) / Def2-SVP level for the DFT-optimised ground-state (52-78) structure.

Coupled states	(52-60)	(52-78)	(52-90)
$\langle [D_0T_1] \vert \hat{H} \vert [D_1S_0] \rangle$	-1	-3	4
$\langle [D_0T_1] \vert \hat{H} \vert [CT] \rangle$	54	-10	-1
$\langle [D_1S_0] \vert \hat{H} \vert [CT] \rangle$	251	96	-1

Table S13: Electronic couplings (in meV) between the $^2[D_1S_0]$, $^2[D_0T_1]$ and ^2CT diabatic states as a function of the Cz-An torsion angle computed at RAS-CI(10,11) / Def2-SVP level.

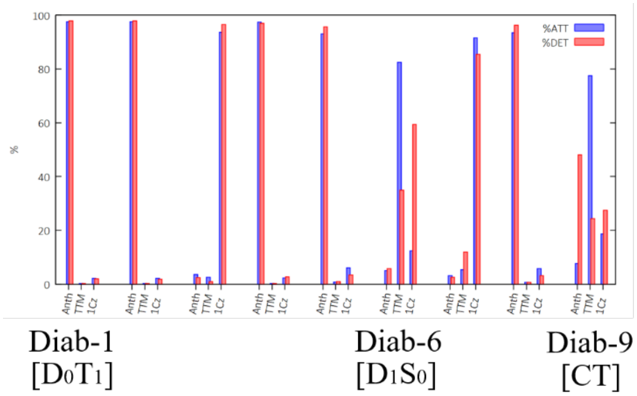

Diab. En.		Anth	TTM	1Cz
$[D_0T_1]$ (4.053 eV)	**% ATT**	97.5	0.3	2.2
	**% DET**	97.8	0.2	2.0
$[D_1S_0]$ (5.090 eV)	**% ATT**	5.2	82.4	12.4
	**% DET**	5.8	34.9	59.3
[CT] (5.665 eV)	**% ATT**	3.7	77.5	18.8
	**% DET**	48.1	24.4	27.5

Figure S26: Attachment and detachment density analysis for the DFT-optimised ground-state (52-78) structure.

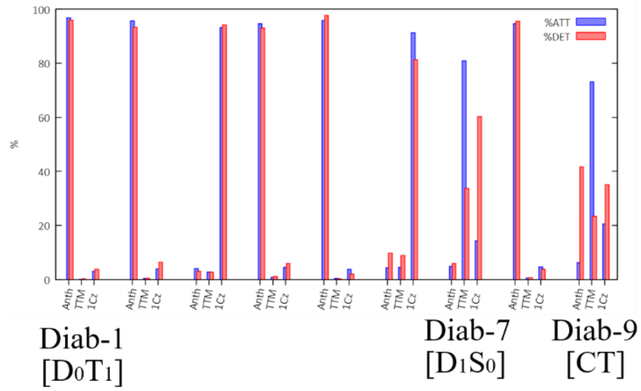

Diab. En.		Anth	TTM	1Cz
$[D_0T_1]$ (3.270 eV)	**% ATT**	96.8	0.17	3.0
	**% DET**	95.9	0.18	3.8
$[D_1S_0]$ (5.208 eV)	**% ATT**	4.9	80.8	14.3
	**% DET**	5.9	33.8	60.3
[CT] (5.529 eV)	**% ATT**	6.2	73.2	20.6
	**% DET**	41.6	23.4	35.1

Figure S27: Attachment and detachment density analysis for the (52-60) conformer.

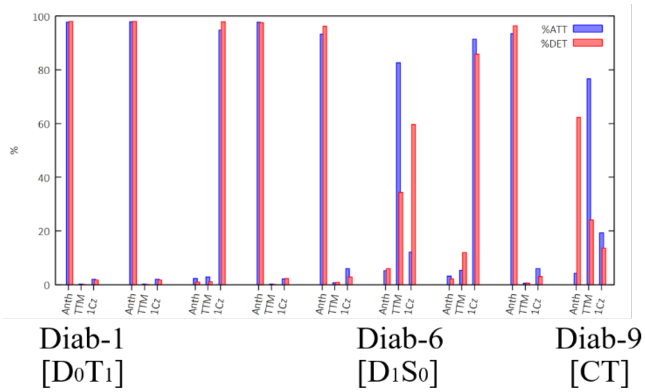

Diab. En.		Anth	TTM	1Cz
$[D_0T_1]$ (3.300 eV)	**% ATT**	97.7	0.2	2.0
	**% DET**	98.0	0.2	1.8
$[D_1S_0]$ (5.193 eV)	**% ATT**	5.3	82.7	12.1
	**% DET**	5.9	34.4	56.7
[CT] (5.812 eV)	**% ATT**	4.2	76.6	19.2
	**% DET**	62.3	24.1	13.6

Figure S28: Attachment and detachment density analysis for the (52-90) conformer.

By looking at Table S11 we highlight that, while the magnitude of the coupling between the diabatic state $^2[D_1S_0]$ and ^2CT barely changes by increasing the size of the diabatization, the energy of the diabatic state $^2[D_0T_1]$ changes significantly, in particular moving from the 3x3 to the 9x9. This variation might come from interactions between the diabatic states localised on the An core. To address this, a partial diagonalization of the anthracene block of the diabatic Hamiltonian was done as follows:

1) we built the An block-Hamiltonian containing the energy and electronic couplings between the diabatic states localised on the An, *i.e.,* diabatic states number 1, 2, 4, 5 and 8 (Figure S26);

$$H_{An} = \begin{bmatrix} \varepsilon_1^{An} & V_{12}^{An} & V_{13}^{An} & \cdots & V_{15}^{An} \\ V_{12}^{An} & \varepsilon_2^{An} & V_{23}^{An} & \cdots & V_{25}^{An} \\ V_{13}^{An} & V_{23}^{An} & \varepsilon_3^{An} & \cdots & V_{35}^{An} \\ \vdots & \vdots & \vdots & \ddots & \vdots \\ V_{15}^{An} & V_{25}^{An} & V_{35}^{An} & \cdots & \varepsilon_5^{An} \end{bmatrix}$$

2) we diagonalised the block-Hamiltonian to obtain the unitary matrix U_{An};

$$\overline{H}_{An} = U_{An}^{\dagger} H_{An} U_{An}$$

3) since the $^2[D_1S_0]$ - $^2[D_0T_1]$ coupling is potentially affected by the partial diagonalization in the An block, we applied a similar unitary transformation to the (An)-(TTM-1Cz) part of the diabatic Hamiltonian, *i.e.,* a vector containing the electronic couplings between the diabatic state localised on the An and the diabatic state $^2[D_1S_0]$. Note that the matrix $U_{TTM-1Cz}$ reduces to 1, since in the diabatization procedure only one diabatic state localised on the TTM-1Cz moiety was obtained.

$$\overline{H}_{An-TTM-1Cz} = U_{An}^{\dagger} \begin{bmatrix} V_1^{An-TTM-1Cz} \\ V_2^{An-TTM-1Cz} \\ V_3^{An-TTM-1Cz} \\ \vdots \\ V_5^{An-TTM-1Cz} \end{bmatrix} U_{TTM-1Cz}$$

Similarly, the $^2[D_1S_0]$ - ^2CT coupling could also be affected by the partial diagonalization on the An block. A similar unitary transformation was also applied to the An ^2CT block of the diabatic Hamiltonian.

	Before partial diagonalization		***After partial diagonalization***	
	$\langle [D_0T_1] \vert \hat{H} \vert [D_1S_0] \rangle$	$\langle [D_0T_1] \vert \hat{H} \vert [CT] \rangle$	$\langle [D_0T_1] \vert \hat{H} \vert [D_1S_0] \rangle$	$\langle [D_0T_1] \vert \hat{H} \vert [CT] \rangle$
(52-60)	-1	54	-1	54
(52-78)	-3	-10	4	13
(52-90)	4	-1	-5	2

Table S14: Electronic couplings (in meV) between $^2[D_0T_1]$ and $^2[D_1S_0]$, ^2CT before and after the partial diagonalization.

Overall, the partial diagonalization does not bring any significant changes in the magnitude of the electronic couplings involving the $^2[D_0T_1]$ state, validating the results obtained previously and thus ensuring the reliability of the 9x9 diabatization.

(iv) Kinetic model

Excited state dynamics was simulated using a kinetic model assuming two different scenarios. The first one entails a two-step process, where the energy transfer between $^2[D_1S_0]$ and $^2[D_0T_1]$ is actively mediated by the ^2CT state. The second one assumes a superexchange mechanism, where the $^2[D_1S_0]$ - $^2[D_0T_1]$ direct conversion is mediated by a virtual ^2CT state. The two processes can be described by the following kinetic equations:

Two-step process

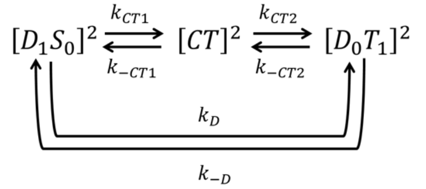

Superexchange

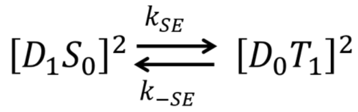

For each elementary process, the rate constant was calculated using the Marcus-Levich-Jortner equation using the energy differences computed at the CAS(9,7) + NEVPT2 level and electronic couplings computed with the 9x9 diabatization. The inner reorganization energy λ_i was computed quantum-mechanically by summing the relaxation energies of the fragments involved in process. For example, the reorganization energy of the $^2CT \rightarrow {}^2[D_0T_1]$ process was computed summing the relaxation energy of the $TTM^{(-)} \rightarrow TTM$ and $An^{(+)} \rightarrow {}^3An$ processes, as obtained at the DFT level (ωB97X-D/6-31G(d,p)). The outer reorganization energy λ_s was set at 0.1 eV and the effective frequency at 0.15 eV.

In the superexchange scenario, the interaction between $^2[D_1S_0]$ and $^2[D_0T_1]$ involves an effective coupling computed within a perturbative treatment:

$$V_{eff} = V_0 + \frac{V_{i-CT}V_{CT-f}}{(E_i + E_f)/2 - E_{CT}}$$

where V_0 is the electronic coupling between $^2[D_1S_0]$ and $^2[D_0T_1]$, V_{i-CT} between $^2[D_1S_0]$ and ^2CT, V_{CT-f} between $^2[D_0T_1]$ and ^2CT, while E_i, E_f and E_{CT} are the excitation energies of $^2[D_1S_0]$, $^2[D_0T_1]$ and ^2CT, respectively, computed at the CAS(9,7) + NEVPT2 level.

For the two-step and superexchange mechanisms, the following kinetic coupled differential equations were obtained:

Two-step process

$$\frac{d[D_1S_0]}{dt} = -k_{CT1}[D_1S_0] + k_{-CT1}[CT] - k_D[D_1S_0] + k_{-D}[D_0T_1]$$
$$\frac{d[CT]}{dt} = k_{CT1}[D_1S_0] - k_{-CT1}[CT] - k_{CT2}[CT] + k_{-CT2}[D_0T_1]$$
$$\frac{d[D_0T_1]}{dt} = k_{CT2}[CT] - k_{-CT2}[D_0T_1] + k_D[D_1S_0] - k_{-D}[D_0T_1]$$

which can be written in a matrix form:

$$\begin{bmatrix} \frac{d[D_1S_0]}{dt} \\ \frac{d[CT]}{dt} \\ \frac{d[D_0T_1]}{dt} \end{bmatrix} = \begin{bmatrix} -(k_{CT1} + k_D) & k_{-CT1} & k_{-D} \\ k_{CT1} & -(k_{-CT1} + k_{CT2}) & k_{-CT2} \\ k_D & k_{CT2} & -(k_{-CT2} + k_{-D}) \end{bmatrix} \begin{bmatrix} [D_1S_0] \\ [CT] \\ [D_0T_1] \end{bmatrix}$$

Superexchange

$$\frac{d[D_1S_0]}{dt} = -k_{SE}[D_1S_0] + k_{-SE}[D_0T_1]$$
$$\frac{d[D_0T_1]}{dt} = k_{SE}[D_1S_0] - k_{-SE}[D_0T_1]$$

which can be written in a matrix form:

$$\begin{bmatrix} \frac{d[D_1S_0]}{dt} \\ \frac{d[D_0T_1]}{dt} \end{bmatrix} = \begin{bmatrix} -k_{SE} & k_{-SE} \\ k_{SE} & -k_{-SE} \end{bmatrix} \begin{bmatrix} [D_1S_0] \\ [D_0T_1] \end{bmatrix} \leftrightarrow \frac{d[\boldsymbol{C}]}{dt} = \boldsymbol{K}[\boldsymbol{C}]$$

where $[\boldsymbol{C}]$ is the vector containing the population of the excited states at time *t* and $\boldsymbol{K}$ is the matrix containing the rate constants. The general solution of these differential equations reads: $[\boldsymbol{C}](t) = [\boldsymbol{C}]_0 exp(\boldsymbol{K}t)$, where $[\boldsymbol{C}]_0$ is the vector containing the initial population of the excited states at time $t = 0$. To solve the differential equations a numerical integration was carried out employing a Taylor expansion of the exponential term up to the second order:

$$[\boldsymbol{C}](\Delta t) = [\boldsymbol{C}]_0(\boldsymbol{I} + \boldsymbol{K}\Delta t + \frac{1}{2}\boldsymbol{K}^2\Delta t^2)$$

	CT1	*CT2*	*-CT2*	*-CT1*	*D*	*-D*	*SE*	*-SE*
ΔE [eV]	0.06	-0.08	0.08	-0.06	-0.02	0.02	-0.02	0.02
V_{el} [meV] (52-60)<br>V_{el} [meV] (52-78)<br>V_{el} [meV] (52-90)	251<br>96<br>1	54<br>10<br>2	54<br>10<br>2	251<br>96<br>1	1<br>3<br>5	1<br>3<br>5	27<br>11<br>4	27<br>10<br>4
λ_i [eV]	0.33	0.24	0.30	0.38	0.60			
λ_s [eV]	0.10							
$\hbar\omega_{\text{eff}}$ [eV]	0.15							
κ [s^{-1}] (52-60)<br>κ [s^{-1}] (52-78)<br>κ [s^{-1}] (52-90)	3.0×10^{13}<br>4.4×10^{12}<br>3.9×10^{8}	3.4×10^{13}<br>1.2×10^{12}<br>2.9×10^{10}	9.1×10^{11}<br>3.1×10^{10}<br>7.9×10^{8}	2.5×10^{14}<br>3.7×10^{13}<br>3.2×10^{9}	2.7×10^{8}<br>3.7×10^{9}<br>1.5×10^{10}	1.2×10^{8}<br>1.6×10^{9}<br>6.6×10^{9}	4.0×10^{11}<br>6.2×10^{10}<br>7.0×10^{9}	1.8×10^{11}<br>2.8×10^{10}<br>3.1×10^{9}

Table S15: CAS(9,7) + NEVPT2 energy difference (ΔE), electronic coupling (V_{el}), inner (λ_i) and outer (λ_s) reorganization energy, effective frequency ($\hbar\omega_{\text{eff}}$) introduced in the Marcus-Levich-Jortner equation and resulting rate constant (κ) for the elementary process of the two-step and superexchange mechanism.

Starting with the two-step process, we assessed the population variation by changing the initial conditions, specifically by setting the $^2[D_0T_1]$ ($^2[D_1S_0]$) initial population equal to 0.0 (1.0), 0.1 (0.9), 0.2 (0.8) and 0.3 (0.7). The crossing between the $^2[D_1S_0]$ and $^2[D_0T_1]$ state occurs at shorter timescales compared to the case where the $^2[D_1S_0]$ population is set to 1.0 (Figure S29, Table S16). In all cases, we reach a saturation regime after 20 ps with $^2[D_1S_0]$ = 0.20, $^2[D_0T_1]$ = 0.77 and ^{2}CT = 0.03. Nevertheless, by fitting the $^2[D_1S_0]$ population variation with a bi-exponential function, the resulting rate of the process remain constant to $1.6\cdot10^{11}$ s^{-1}, with a lifetime of 6 ps. It is worth noting that in all cases the population of the ^{2}CT state undergoes a rapid increase within the first 0.1 ps to gradually decrease until 20 ps, reaching the constant value of 0.03.

Considering the (52-60) conformer, the larger values of the electronic couplings involving the ^{2}CT state (Table S15), results in larger rates for the CT elementary processes, with respect to the equilibrium geometry which leads to an energy transfer process on the sub-picosecond timescale (Figure S29(a), Table S16). Regarding the (52-90) conformer, the drastic decrease of the magnitude of the electronic couplings associated with the CT process (Table S14), leads their rate constants to be significantly smaller than at the equilibrium geometry. As a result, the timescale of the energy transfer process becomes the longest (45 ps), with the crossing occurring between 60 ps and 30 ps, depending on the initial conditions.

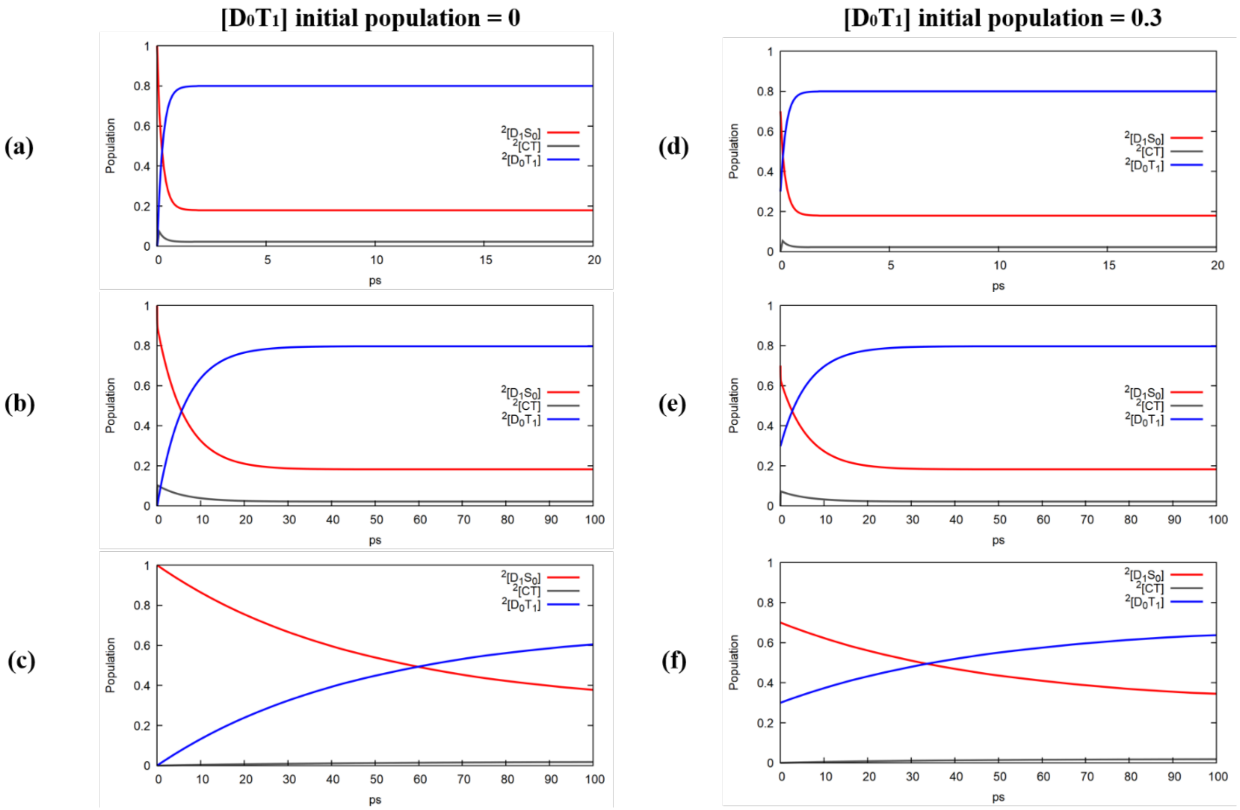

Figure S29: ^{2}[D$_1$S$_0$], ^{2}[D$_0$T$_1$] and ^{2}CT population variation as a function of time by setting the initial population of ^{2}[D$_0$T$_1$] either to 0 or 0.3 for the (52-60) (a)/(d), (52-78) (b)/(e) and (52-90) (c)/(f) conformer.

	Initial conditions	Crossing time [ps]	a	b [s^{-1}]	c	d [s^{-1}]	τ [ps]
(52-60)	$[D_1S_0]^2 = 1.0$ $[D_0T_1]^2 = 0.0$	0.1	0.80 (0.69)	4.5×10^{12} (1.4)	0.19 (1.5)	3.1×10^{8} (49.6)	0.2
	$[D_1S_0]^2 = 0.9$ $[D_0T_1]^2 = 0.1$	0.1	0.71 (0.12)	4.4×10^{12} (0.22)	0.18 (0.03)	1.0×10^{7} (54.0)	0.2
	$[D_1S_0]^2 = 0.8$ $[D_0T_1]^2 = 0.2$	0.1	0.61 (0.13)	4.5×10^{12} (0.22)	0.18 (0.03)	9.0×10^{8} (54.1)	0.2
	$[D_1S_0]^2 = 0.7$ $[D_0T_1]^2 = 0.3$	0.1	0.51 (0.13)	4.5×10^{12} (0.24)	0.18 (0.03)	7.6×10^{8} (54.3)	0.2
(52-78)	$[D_1S_0]^2 = 1.0$ $[D_0T_1]^2 = 0.0$	5.5	0.72 (0.04)	1.6×10^{11} (0.09)	0.18 (0.17)	3.1×10^{8} (16.8)	6.1
	$[D_1S_0]^2 = 0.9$ $[D_0T_1]^2 = 0.1$	4.9	0.600 (0.02)	1.6×10^{11} (0.04)	0.20 (0.03)	7.3×10^{7} (5.4)	6.2
	$[D_1S_0]^2 = 0.8$ $[D_0T_1]^2 = 0.2$	4.0	0.51 (0.02)	1.6×10^{11} (0.04)	0.20 (0.02)	4.3×10^{7} (7.9)	6.2
	$[D_1S_0]^2 = 0.7$ $[D_0T_1]^2 = 0.3$	2.8	0.42 (0.02)	1.6×10^{11} (0.04)	0.20 (0.02)	1.5×10^{7} (19.6)	6.3
(52-90)	$[D_1S_0]^2 = 1.0$ $[D_0T_1]^2 = 0.0$	60	0.67 (0.08)	2.2×10^{10} (0.06)	0.33 (0.16)	7.7×10^{8} (1.4)	45
	$[D_1S_0]^2 = 0.9$ $[D_0T_1]^2 = 0.1$	53	0.57 (0.06)	2.2×10^{10} (0.05)	0.32 (0.12)	7.2×10^{8} (1.1)	45
	$[D_1S_0]^2 = 0.8$ $[D_0T_1]^2 = 0.2$	44	0.48 (0.05)	2.2×10^{10} (0.04)	0.32 (0.08)	5.8×10^{8} (0.94)	45
	$[D_1S_0]^2 = 0.7$ $[D_0T_1]^2 = 0.3$	33	0.40 (0.10)	2.1×10^{10} (0.08)	0.30 (0.13)	1.5×10^{8} (6.0)	47

Table S16: Crossing time and parameters obtained by fitting the $^2[D_1S_0]$ population decay employing a bi-exponential function ($P_{[D_1S_0]^2}(t) = a \cdot exp(-bt) + c \cdot exp(-dt)$). For each coefficient value, the associated percentage error is reported (in parenthesis). The time constant τ in the last column is the result of $\frac{1}{b}$.

We also assessed the role played by the direct and CT processes by considering two scenarios: (i) neglecting the direct $^2[D_1S_0]$ - $^2[D_0T_1]$ conversion (*i.e.,* setting $k_D = k_{-D} = 0$); (ii) neglecting the processes involving the CT state (*i.e.* setting $k_{CT1} = k_{-CT1} = k_{CT2} = k_{-CT2} = 0$). As shown in Figure S30(b), neglecting the direct conversion process for the case of the (52-78) conformer has no impact on the population variation of the three states, as expected due to the smaller value of the rate constants of the direct processes with respect to those involving the ^{2}CT state. On the other hand, neglecting the CT processes leads to a much longer timescale, with the crossing between the $^2[D_1S_0]$ and $^2[D_0T_1]$ population occurring beyond 100 ps. Thus, the involvement of the ^{2}CT state is essential to ensure an energy transfer within ps timescales. Similarly, the CT processes govern the energy transfer for the (52-60) conformer, thanks to the significantly larger rates with respect to the direct processes (Figure S30(a)). An opposite trend is instead obtained for the (52-90) conformer: the larger values of the rates associated with the direct process with respect to the CT elementary processes lead the energy transfer to be governed by the former. As shown in Figure S30(c), by neglecting the direct process the crossing between $^2[D_1S_0]$ and $^2[D_0T_1]$ population occurs beyond 100 ps, with the two populations slowly approaching each other. Instead, by neglecting the CT processes the population variation recovers the one shown in Figure S29, with the crossing occurring at 60 ps.

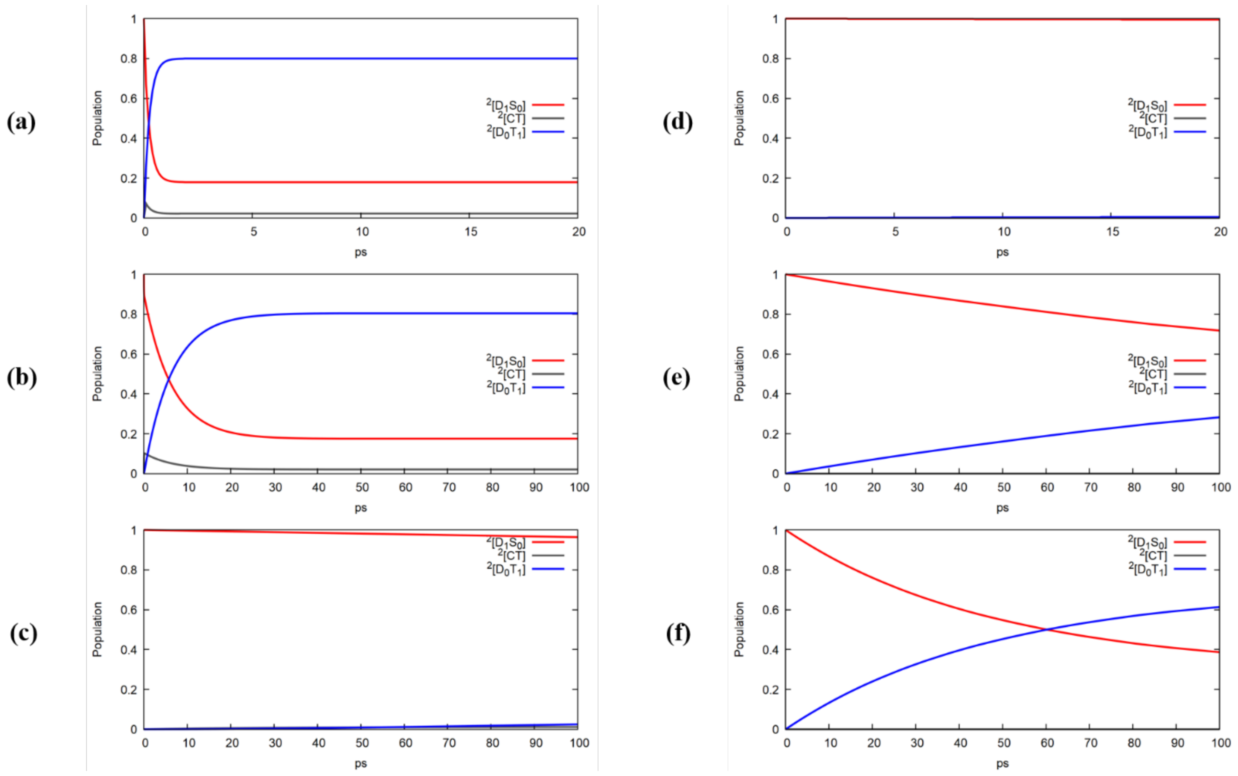

Figure S30: $^2[D_1S_0]$, $^2[D_0T_1]$ and ^{2}CT population variation as a function of time within the two-step mechanism, neglecting either the direct or the CT processes for the (52-60) (a)/(d), (52-78) (b)/(e) and (52-90) (c)/(f) conformer.

We shall now consider the second scenario, where the ^{2}CT state participates as a virtual state in a superexchange-like mechanism.

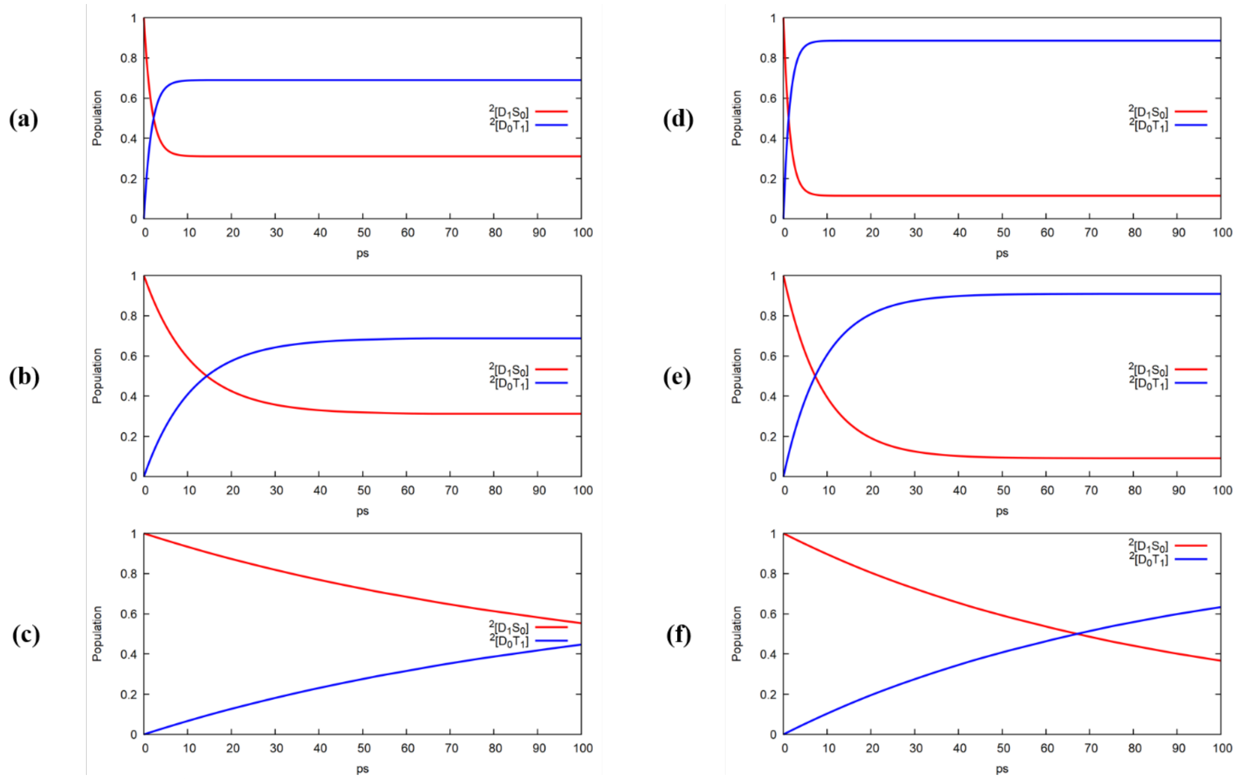

Figure S31: ^{2}[D$_1$S$_0$] and ^{2}[D$_0$T$_1$] population variation as a function of time within the superexchange mechanism. The energy difference ΔE was set either to 0.02 or 0.05 eV for the (52-60) (a)/(d), (52-78) (b)/(e) and (52-90) (c)/(f) conformer.

As shown in Figure S31(b), for the (52,78) conformer the crossing between the ^{2}[D$_1$S$_0$] and ^{2}[D$_0$T$_1$] population occurs at 15 ps, and the fitting with the bi-exponential function results in a total rate of $9.0 \cdot 10^{10}$ s^{-1}, giving a lifetime of 11 ps (Table S17), which is longer than the one obtained with the two-step scenario but still comparable with the experiment.

Interestingly, by slightly increasing ΔE to 0.05 eV we induced a stronger asymmetry in the rate constant values, with k_{SE} becoming larger than k_{-SE}. As a result, the ^{2}[D$_1$S$_0$] → ^{2}[D$_0$T$_1$] process is favoured, leading the crossing between the two populations occurring at 7 ps and a ratio (R) between the ^{2}[D$_0$T$_1$] and ^{2}[D$_1$S$_0$] in the saturation regime increasing from 2.2 to 9.9 (Figure S31(e) and Table S17). Yet, the timescale of the entire process remains similar, with the lifetime slightly decreasing to 9 ps, suggesting that the variation of the energy gap between the states does not affect the timescales of the process. The same trend is observed for the (52-60) conformer, with the process occurring at shorter timescale thanks to the higher value of the effective couplings, in turn resulting in higher rates. On the contrary, the smaller effective couplings characterizing the (52-90) conformer lead to longer process than the previous cases.

	ΔE [eV]	V_{eff} [meV]	k_{SE} $[s^{-1}]$	k_{-SE} $[s^{-1}]$	a	b $[s^{-1}]$	c	d $[s^{-1}]$	τ [ps]	R
(52-60)	0.02	*27*	4.0×10^{11}	1.8×10^{11}	0.69	5.8×10^{11}	0.31	1.4×10^{7}	1.7	2.2
	0.05	27	6.2×10^{11}	8.0×10^{10}	0.89	7.0×10^{11}	0.11	-1.5×10^{7}	1.4	7.7
(52-78)	0.02	11	6.2×10^{10}	2.8×10^{10}	0.69	9.0×10^{10}	0.31	1.1×10^{8}	11	2.2
	0.05	11	1.0×10^{11}	1.0×10^{10}	0.69	1.1×10^{11}	0.31	1.1×10^{8}	9	9.9
(52-90)	0.02	4	7.0×10^{9}	3.1×10^{9}	0.42	1.3×10^{10}	0.58	2.8×10^{9}	77	2.4
	0.05	4	1.1×10^{10}	1.4×10^{9}	0.17	2.3×10^{10}	0.83	8.6×10^{9}	43	1.7

Table S17: CAS(9,7) + NEVPT2 energy difference (ΔE), effective coupling (V_{eff}), forward (k_{SE}) and backward (k_{-SE}) rate, parameters obtained by fitting the $^2[D_1S_0]$ population decay employing a bi-exponential function ($P_{[D_1S_0]^2}(t) = a \cdot exp(-bt) + c \cdot exp(-dt)$), the time constant τ and $^2[D_0T_1]/^2[D_1S_0]$ ratio (R) at 100 ps.

Overall, the kinetic simulations revealed that the involvement of the ^{2}CT state, either as an active or a virtual state, is necessary to obtain an energy transfer process occurring in a few ps timescales. However, the limitations of the theoretical framework do not allow to discriminate whether one mechanism or the other is the most relevant.

Supplementary References:

46. Abdurahman, A. *et al.* Understanding the luminescent nature of organic radicals for efficient doublet emitters and pure-red light-emitting diodes. *Nat. Mater.* **19**, 1224–1229 (2020).
47. Gélinas, S. *et al.* The Binding Energy of Charge-Transfer Excitons Localized at Polymeric Semiconductor Heterojunctions. *J. Phys. Chem. C* **115**, 7114–7119 (2011).
48. Jeschke, G. *et al.* DeerAnalysis2006—a comprehensive software package for analyzing pulsed ELDOR data. *Appl. Magn. Reson.* **30**, 473–498 (2006).
49. Köhler, A. & Beljonne, D. The Singlet–Triplet Exchange Energy in Conjugated Polymers. *Adv. Funct. Mater.* **14**, 11–18 (2004).
50. Lunghi, A. & Totti, F. DFT magnetic characterization of a Fe4 SMMs series: from isotropic exchange interactions to multi-spin zero field splitting. *J. Mater. Chem. C* **2**, 8333–8343 (2014).
